# In Situ UV Torsional Force Spectroscopy for Real‐Time Mapping of Cross‐linking Kinetics and Mechanical Properties in Polymeric Films

**DOI:** 10.1002/smtd.202502210

**Published:** 2026-03-22

**Authors:** Marvin Hoffer, Felix Petersein, Martin Dehnert, Tobias Andreas Lintner, Hikmet Sezen, Jan Philipp Hofmann, Christian Dietz

**Affiliations:** ^1^ Physics of Surfaces Institute of Materials Science Technical University of Darmstadt Darmstadt Germany; ^2^ Chemische Physik Fakultät für Naturwissenschaften Technische Universität Chemnitz Chemnitz Germany; ^3^ Surface Science Laboratory Department of Materials and Geosciences Technical University of Darmstadt Darmstadt Germany

**Keywords:** atomic force microscopy, cross‐linking density, in situ spectroscopy, nanomechanics, polymer films, torsional force spectroscopy, UV cross‐linking

## Abstract

An advanced in situ ultraviolet (UV) torsional force spectroscopy (TFS) technique is introduced for real‐time mapping of in‐plane nanomechanical properties in UV‐cross‐linked poly(1,4‐butadiene) (PB) films on the nanoscale. This approach combines UV illumination and torsional force spectroscopy, offering high‐resolution insights into in‐plane shear stress, storage shear modulus, and dissipated energy throughout thiol‐ene cross‐linking reactions. Complementary swelling experiments provide a direct correlation between increasing UV dose, cross‐linking density, and mechanical stiffness. Ex situ measurements confirm the reliability of this in situ method with only minor deviations attributed to UV scattering effects and experimental variations. The reduced stiffness and increased heterogeneity observed by TFS in PB films containing phase‐separated polystyrene (PS) domains are associated with changes in the spatial distribution of the cross‐linking agent and the resulting mechanical response of the PB matrix, consistent with complementary X‐ray photoelectron spectroscopy (XPS) and Fourier transform infrared reflection absorption spectroscopy (FT‐IRRAS) analyses. Overall, this work establishes in situ UV torsional force spectroscopy as a predictive and quantitative method to optimize UV curing conditions and additive concentrations for tailoring the mechanical properties and performance of polymeric materials.

## Graphical abstract

This study introduces an in situ UV torsional force spectroscopy method to track how polymers harden under UV light. The technique maps stiffness and energy loss beneath the surface in real time. It reveals how cross‐linking develops and identifies regions with different mechanical behavior, helping optimize UV curing processes in polymer materials.

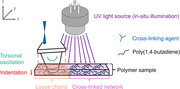

## 1 Introduction

Polybutadiene is a widely used synthetic rubber, valued for its outstanding elasticity and used in diverse applications ranging from safety‐critical car tires [1] and rocket propellants [2] to sports equipment [3], adhesives [4], and numerous industrial products. Its hardness, stiffness, and strength can be tuned by cross‐linking the polymer chains through ultraviolet (UV) radiation in combination with additives such as photoinitiators and cross‐linking agents [5, 6]. However, optimizing the additive concentrations remains a major challenge: inappropriate levels may cause instabilities, phase separation, or unfavorable mechanical properties. In safety‐critical components such as car tires, deficiencies may compromise safety and ultimately endanger human lives. Consequently, the ability to monitor and understand cross‐linking reactions in polymer–additive systems, particularly at the nanoscale, is essential. Moreover, the cross‐linking density is the central parameter for characterizing these reactions, and its precise control is crucial across technological applications. For instance, in flexographic printing, scattering of UV light during exposure of photosensitive resists can induce cross‐linking gradients that reduce printing quality [7]. In medicine, hydrogels rely on finely tuned cross‐linking to achieve controlled and localized drug release, where inadequate adjustment may cause severe side effects [8]. In electronics, block copolymer‐based transistors require optimized cross‐linking to balance mechanical flexibility, solvent resistance, and electrical conductivity [9]. The determination of cross‐linking density depends strongly on the observation scale. At the macroscale, it can be assessed using conventional techniques such as solvent swelling (Flory–Rehner theory) [10], stress–strain analysis (Mooney–Rivlin theory) [11, 12], rheology [13], dynamic mechanical analysis (DMA) [14], and nuclear magnetic resonance (NMR) [15]. At the micro‐ to nanoscale, atomic force microscopy (AFM) has proven to be a powerful method for probing polymer mechanics [16, 17, 18, 19, 20, 21, 22, 23, 24, 25, 26, 27, 28]. AFM‐based static force spectroscopy, in combination with rubber elasticity theory [29] and indentation models [30], has enabled correlations between cross‐linking density and storage shear modulus [31, 32]. Furthermore, advanced AFM mechanical mapping methods, especially PeakForce quantitative nanomechanical mapping (PFQNM), can map quantitative mechanical properties of polymeric materials at the nanoscale, such as stiffness, adhesion, and deformation [33, 34, 35]. However, these AFM approaches are restricted to out‐of‐plane measurements and fail to directly correlate cross‐linking density with the in‐plane mechanical properties governed by the shear modulus.

In this study, we introduce an in situ UV torsional force spectroscopy (TFS) mode, building upon the method of Walter et al. [36], which enables the determination of in‐plane nanomechanical properties of polybutadiene films under UV illumination. This technique provides access to mechanical quantities, such as in‐plane shear stress and dissipated energy, as functions of indentation depth. Importantly, it also enables depth‐resolved analysis of the evolving cross‐linking behavior during UV illumination, allowing spatially resolved quantification of stiffness gradients and network formation throughout the film thickness. These nanoscopic results are verified with ex situ torsional force spectroscopy and correlated with macroscopic swelling measurements, thereby offering a multiscale perspective on cross‐linking density. Because this spectroscopy technique relies on force–distance curves, out‐of‐plane mechanical properties are inherently captured. Moreover, we propose a model of the AFM tip interactions with non‐cross‐linked and UV‐cross‐linked polymer chains, providing mechanistic insights into cross‐linking at the micro‐/nanoscale. To investigate the impact of heterogeneity on the mechanical properties, polystyrene (PS) was added as an immiscible secondary polymer, forming phase‐separated domains that perturb the UV‐induced cross‐linking of the polybutadiene matrix. Overall, this framework establishes a foundation for optimizing polymer–additive formulations and mitigating defect formation. Beyond fundamental understanding, this approach provides a pathway toward in situ process optimization by enabling real‐time adjustment of UV intensity, exposure time, and curing sequence—ultimately reducing experimental iterations and production costs in the development of advanced polymeric coatings and composites.

## 2 Results and Discussion

### 2.1 In‐Plane Nanomechanical Mapping of Poly(1,4‐butadiene) (PB) Films and Correlation with Cross‐Linking Density

**FIGURE 1:**
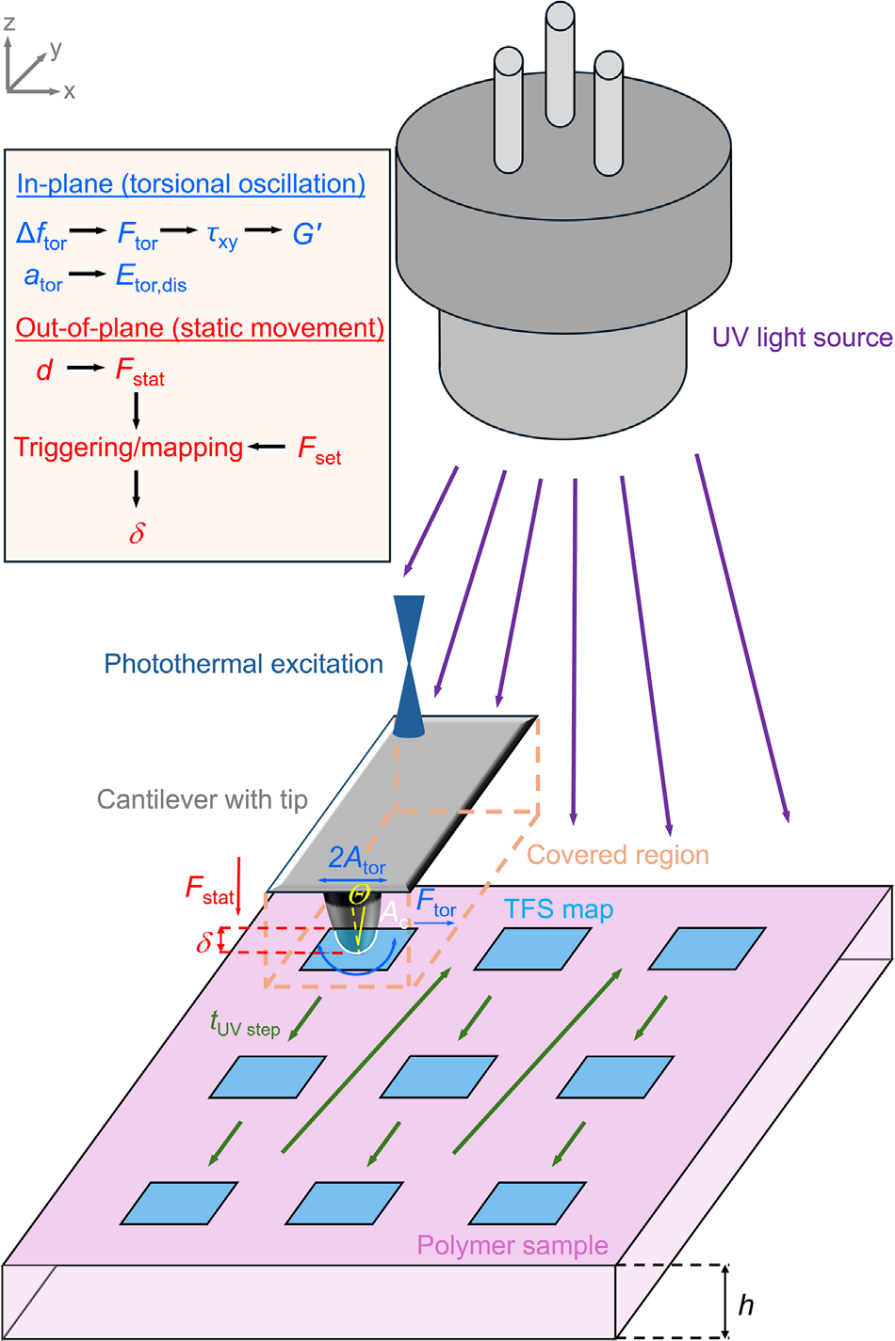
Excitation and detection scheme of the in situ UV torsional force spectroscopy mode for investigating the subsurface nanomechanical properties of the cross‐linked PB films. In this figure, the vertical cantilever deflection is denoted by *d*, vertical static force by *F*_stat_, set point force by *F*_set_, indentation depth of the AFM tip by *δ*, half‐cone angle of the tip by *Θ*, tip–sample contact area by *A*_c_, torsional oscillation amplitude by *A*_tor_, torsional tip–sample force by *F*_tor,_ measurement duration of one torsional force spectroscopy map by *t*_UV step_, polymer film thickness by *h*, torsional frequency shift by Δ*f*_tor_, in‐plane shear stress by *τ*_xy_, storage shear modulus by *G*’, torsional excitation amplitude by *a*_tor_, and torsional dissipated energy by *E*_tor,dis_.

We developed an in situ UV torsional force spectroscopy mode, extending the approach from Walter et al. [36], to map the in‐plane nanomechanical behavior of UV‐cross‐linked PB films (see Figure 1). The general working principle of torsional force spectroscopy has been previously described in detail [37]. Here, we focus on its extension to include in situ UV illumination. In the following paragraph, we briefly summarize the functional principle of torsional force spectroscopy.

During each *z*‐cycle, the cantilever quasi‐statically approaches and retracts from the PB film surface. The vertical deflection signal is used as the feedback parameter, where a set point force *F*_set_ regulates the indentation depth *δ* of the tip into the film. Once *F*_set_ is reached during approach, the maximum indentation depth is attained. Unlike conventional static force spectroscopy, the cantilever is simultaneously driven into torsional oscillations by photothermal actuation. This actuation is realized by focusing a power‐modulated laser onto the cantilever base, positioned a few micrometers off the longitudinal centerline. The cantilevers used in this study comprised a silicon body coated with a thin gold layer. When the laser illuminates the gold‐coated backside, localized periodic heating occurs, and due to the mismatch in thermal expansion coefficients between silicon and gold, torsional eigenmodes are excited. Owing to the small amplitude of 0.75 nm of these oscillations relative to the tip height, the tip apex motion can be approximated as parallel to the film surface, with lateral forces only occurring during indentation. The torsional response of the cantilever was detected through the lateral photodiode signal and monitored with a phase‐locked loop, which measured the torsional frequency shift Δ*f*_tor_ and adjusted excitation amplitude *a*_tor_ to maintain the oscillation amplitude with a proportional‐integral (PI) feedback loop. By mapping the surface, in‐plane nanomechanical volume properties could be quantified with high spatial resolution on Δ*f*_tor_(*x*,*y*,*z*) and *a*_tor_(*x*,*y*,*z*).

A particular feature of the in situ UV torsional force spectroscopy configuration is that the cantilever acts as a mask, shielding regions of the PB film directly underneath the cantilever body during data collection (see orange dashed lines in Figure 1), while the surrounding area continues to be irradiated. This ensures that each force map is acquired within a well‐defined UV illumination interval, providing sufficient statistical robustness for the measured in‐plane properties. The number of force maps and the minimum spacing between adjacent maps are limited by the ratio between the maximum scan area (100 × 100 µm^2^) and the cantilever width (approximately 30 µm in this case). Nine consecutive force maps were acquired, each at a different UV illumination interval, in a sawtooth‐like arrangement (see the dark green arrows in Figure 1). This geometry prevents illumination of one mapping area from influencing subsequently measured maps. The first acquired force map corresponds to the pristine state of the PB film. The acquisition time for one force map defines the UV illumination time interval *t*_UV step_. Automated queueing in the software enabled consecutive force map acquisition without interruption. The thiol‐ene reaction mechanism is shown in Figure S1, and the setup of the in situ UV torsional force spectroscopy mode is illustrated in Figure S2 of the Supporting Information.

In our recent study [37], we derived an analytical expression for the torsional tip–sample force *F*_tor_. This force is related to the in‐plane tip–sample force constant *k*_ip_, the torsional oscillation amplitude *A*_tor_, the torsional frequency shift Δ*f*_tor_, and the resonance frequency of the torsional eigenmode *f*_0,tor_, as given in Equation (1).

(1) $$F_{\mathrm{tor}}\left(\delta \right)=\frac{4k_{\mathrm{ip}}A_{\mathrm{tor}}\Delta f_{\mathrm{tor}}}{f_{0,\mathrm{tor}}}\;$$

The in‐plane shear stress *τ*_xy_ is obtained by dividing *F*_tor_ by the tip–sample contact area *A*_c_ according to Equation (2), where *F*_shear_ is the shear force and *A* the sheared area [37].

(2) $$\tau_{\mathrm{xy}}=\frac{F_{\mathrm{shear}}}{A}=\frac{F_{\mathrm{tor}}}{A_{c}}\;$$

For simplicity, the AFM tip is approximated as a spherical indenter with conical side walls. Accordingly, two regimes of the tip–sample contact area must be differentiated depending on the indentation depth of the tip [37]. The shell surface is used instead of the projected area, since the polymer chains surrounding the entire tip are involved in the shearing interaction during the lateral tip motion. Therefore, the shell surface of a sphere is taken into account for the case that the indentation depth *δ* is lower than or equal to the tip radius *R*, as shown in Equation (3).

(3) $$A_{c}=A_{\mathrm{sphere}}=2\pi R\delta ,\;\;for\;\delta \leq R$$

Furthermore, Equation (4) accounts for the sum of the surface areas of the sphere and a truncated cone with a half‐cone angle *Θ* to calculate the tip–sample contact area when the indentation depth exceeds the tip radius.

(4) $$\begin{array}{ccc} A_{c}=A_{\mathrm{sphere}}\;\left(\delta =R\right)+A_{\mathrm{cone}} & = & 2\pi R^{2}+\frac{2\pi R\left(\delta -R\right)}{\cos\Theta }\\ & & +\pi \frac{\tan\Theta }{\cos\Theta }{\left(\delta -R\right)}^{2},\;for\;\delta >R \end{array}$$

In torsional force spectroscopy, the complex shear modulus G* can be expressed according to Equation (5) as a combination of its real component *G*′ and imaginary component *G*″,

(5) $$\;G^{*}=G^{\prime }+iG^{\prime \prime }$$

where *G*′ denotes the storage shear modulus, describing the elastic contribution associated with energy storage during shear deformation, and *G*″ represents the loss shear modulus, reflecting viscous or dissipative processes such as frictional energy losses.

For the present study, we focus on the storage shear modulus *G*′, defined in Equation (6) as the ratio between in‐plane shear stress *τ*_xy_ (derived from Equation (2)) and the shear strain *γ*_xy_.

(6) $$G^{\prime }=\frac{\tau_{\mathrm{xy}}}{\gamma_{\mathrm{xy}}}=\frac{F_{\mathrm{shear}}/A}{\Delta x/l}=\frac{F_{\mathrm{tor}}}{A_{c}}\;\frac{\left(h-\delta \right)}{A_{\mathrm{tor}}}$$

Here, Δ*x* corresponds to the lateral tip displacement, corresponding to the torsional oscillation amplitude *A*_tor_, and *l* the effective sample thickness, defined as the total polymer film thickness *h* (≈100 nm in this work) minus the indentation depth *δ*.

Energy dissipation during the lateral tip motion in each cycle, denoted as *E*_tor,dis_, is quantified based on Equation (7).

(7) $$E_{\mathrm{tor},\mathrm{dis}}=\pi k_{\mathrm{ip}}\;\left(\frac{A_{\mathrm{tor}}A_{0}}{Q_{\mathrm{tor}}}-\frac{A_{\mathrm{tor}}^{2}}{Q_{\mathrm{tor}}}\right)$$

Hereby, *A*_0_ is the torsional free amplitude, and *Q*_tor_ is the quality factor of the torsional eigenmode. The quotient between *A*_0_ and *Q*_tor_ represents the torsional excitation amplitude *a*_tor_ [37].

To explain the procedure linking the raw data measured using torsional force spectroscopy to the absolute in‐plane nanomechanical properties, step‐by‐step guides are shown in Figures S3 and S4 of the Supporting Information.

To correlate the nanoscale mechanical data from in situ UV torsional force spectroscopy with macroscopic cross‐linking behavior, the cross‐linking density *ρ* was calculated using the Flory–Rehner Equation (8): [10, 38]

(8) $$\rho =-\frac{\ln\left(1-\phi \right)+\phi +\chi_{\mathrm{PB}/\mathrm{heptane}}\times \phi^{2}}{V_{m,\mathrm{heptane}}\times \left(\phi^{\frac{1}{3}}-\frac{\phi }{2}\right)}$$

where the swollen polymer volume fraction *φ* is obtained from the areas of floating PB films before (*S*_dry_) and after swelling (*S*_swell_) with n‐heptane:

(9) $$\phi ={\left(\frac{1}{\sqrt{\frac{S_{\mathrm{swell}}}{S_{\mathrm{dry}}}}}\right)}^{3}\;$$

The Flory‐Huggins interaction parameter *χ*_PB/heptane_ for the PB/n‐heptane system is estimated by the relationship in Equation (10) [39].

(10) $$\chi_{\mathrm{PB}/\mathrm{heptane}}=0.428+0.535\times \phi$$

The molar volume *V*_m,heptane_ of n‐heptane as a swelling agent was assumed to be 1.465 × 10^−4^ m^3^ mol^−1^ [39]. The analysis assumes isotropic swelling of the PB films.

**FIGURE 2:**
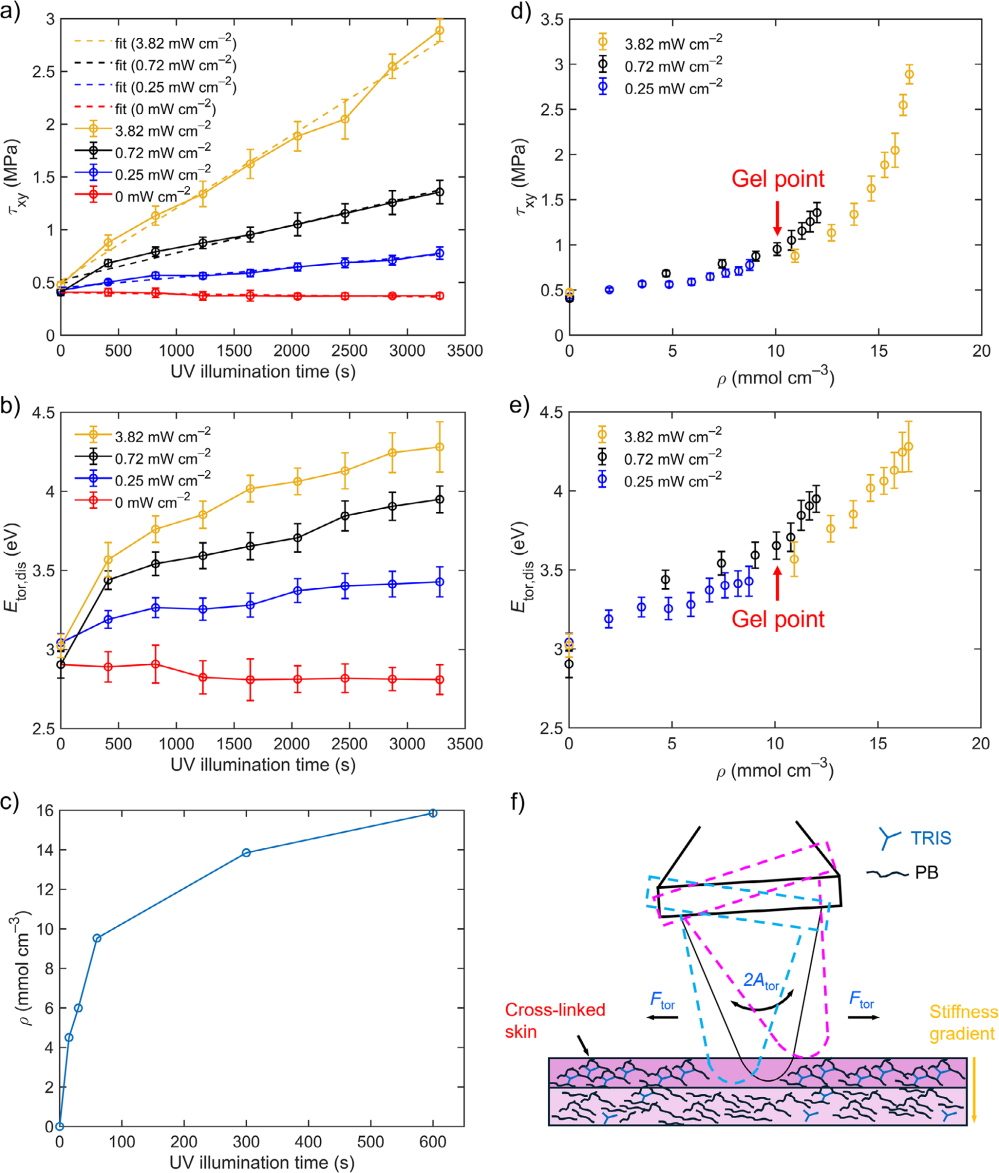
Master curves of (a) in‐plane shear stress and (b) torsional dissipated energy as functions of UV illumination time and intensity for UV cross‐linked PB films at a constant indentation depth of 10 nm. Each data point represents the average of 1024 measurement points from a single force map, and the displayed error bars correspond to the standard deviations (SD) of these 1024 points (number of independent samples: *n* = 1). Therefore, the data of the master curves are presented as mean ± SD. The orange, black, and blue dotted lines in (a) indicate a linear increase of the in‐plane shear stress with increasing UV time. (c) Macroscopic cross‐linking density obtained from swelling experiments as a function of UV illumination time. (d) In‐plane shear stress and (e) torsional dissipated energy as functions of macroscopic cross‐linking density at a constant indentation depth of 10 nm derived from fitting of the macroscopic cross‐linking density as a function of UV dose. The error bars in (d, e) are the same ones as shown in (a, b). The red arrows in (d, e) indicate a possible gel point. (f) Schematic illustration of the interaction between AFM tip and cross‐linked polymer chains during in situ UV torsional force spectroscopy.

Master curves of the in‐plane nanomechanical properties as functions of UV illumination time and at different UV light intensities (see calibration data in Figure S5, Supporting Information) for cross‐linked PB films at a constant tip indentation depth of 10 nm are presented in Figure 2a, b. These curves were obtained using in situ UV torsional force spectroscopy and applying Equations ((1), (2), (3), (4), (7)). Each data point of these curves represents the average of 1024 measurement points from a single force map at the defined UV illumination time. The linear increase of the in‐plane shear stress is indicated in Figure 2a by orange, black, and blue dotted lines. These nanoscale measurements were further correlated, based on the UV dose, with macroscopic cross‐linking densities derived from macroscopic swelling measurements via Equations ((8), (9), (10)) (see Figure 2c and Figure S6 for the patterning procedure of the PB film, Supporting Information). For this purpose, the UV light intensity was converted into UV dose by multiplying it by the respective illumination time used in the in situ UV TFS and swelling experiments. Following this conversion, the macroscopic cross‐linking density as a function of UV dose was fitted with a dual saturation function to account for the presence of different regimes of cross‐linking (see Figure S7 of the Supporting Information for details). This approach allowed us to interpolate missing data points of the macroscopic cross‐linking density required for correlation with the in‐plane nanomechanical quantities. Consequently, the in‐plane shear stress and torsional dissipated energy as functions of macroscopic cross‐linking density were obtained (see Figure 2d, e). Refer to Figures S8 and S9 of the Supporting Information for the curves of the torsional force spectroscopy observables and the mechanical quantities over the full range of indentation depth at different UV light intensities. Possible photothermal effects were investigated by placing a PT100 temperature sensor on a silicon substrate underneath the cantilever holder mounted on the AFM and illuminating the sensor for 1 h with UV light at the maximum intensity used in this study (3.82 mW cm^−2^). A temperature rise by approximately 0.8 °C was measured, indicating that photothermal effects have a negligible influence on the mechanical quantities acquired by in situ UV torsional force spectroscopy.

Both the in‐plane shear stress and the torsional dissipated energy increase with longer UV illumination time and higher intensity (see Figure 2a, b), consistent with the rise in cross‐linking density at higher UV time (see Figure 2c). In contrast, under reference conditions without UV illumination, the mechanical quantities remain nearly constant over the same time period (see red curves in Figure 2a, b). The distribution of the error bars in Figure 2a, b indicates that systematic artifacts caused by tip wear or contamination are unlikely.

The increase in the in‐plane shear stress can be attributed to the thiol‐ene reaction mechanism illustrated in Figure S1 of the Supporting Information. Upon UV illumination, cross‐linking reactions are initiated, resulting in the formation of covalent bonds between PB polymer chains and trimethylolpropane tris(3‐mercaptopropionate) (TRIS) cross‐linking agent molecules. The newly formed covalent bonds act as elastic springs during lateral tip motion, thereby increasing stiffness and simultaneously restricting the mobility of the polymer chains.

The increase in torsional dissipated energy with increasing UV illumination time can be explained by the “inner shielding effect” [40, 41]. Figure 2f illustrates the interaction mechanism between the AFM tip and the cross‐linked polymer network in the torsional force spectroscopy mode: Because the UV light is absorbed within the PB film, the highest light intensity, and therefore the highest radical generation rate, occurs at the surface. The bulk of the PB film is shielded by this cross‐linked skin, so a fully cross‐linked network cannot form instantaneously throughout the film thickness [41]. At early UV illumination times, this effect produces a stiffness gradient through the film. During in situ UV torsional force spectroscopy, the AFM tip shears through layers of different stiffness, and internal friction and energy dissipation are enhanced at the interfaces between stiff and soft regions [42]. As cross‐linking density increases with longer UV illumination, lateral tip motion increasingly occurs via stick–slip and micro‐sliding at these interfaces [42, 43]. Under shear deformation, the mechanical work of the tip is converted into heat, resulting in progressively higher dissipated energy. In addition, covalent bond formation between PB chains and the cross‐linking agent (TRIS) restricts polymer chain mobility, introducing heterogeneous shear deformation within the network [44]. Lateral tip motion then induces irreversible rearrangements of the cross‐linked chains, further increasing internal friction [45]. At sufficiently long UV exposure times, the in‐plane shear stress and torsional dissipated energy saturate as either all available photoinitiator and cross‐linking agent are consumed or a uniform, fully cross‐linked polymer network forms [46].

Figure 2d shows an increase in in‐plane shear stress with increasing cross‐linking density. This trend most likely can be explained by gelation physics and percolation theory. As the cross‐linking density within the PB films rises upon UV illumination, the polymer system undergoes a transition from a liquid‐like to a solid‐like state and reaches the percolation threshold, at which an infinite network spanning over the entire UV‐illuminated PB film area, but not throughout the whole film thickness, is formed for the first time. This transition corresponds to the so‐called gel point [47]. In our case, the gel point occurs at a cross‐linking density of approximately 10 mmol cm^−3^ (see red arrow in Figure 2d), where a change from a linear to an exponential increase can be seen. The torsional dissipated energy resembles the same trend with macroscopic cross‐linking density (see red arrow in Figure 2e). Such nonlinear increases in mechanical properties beyond the gel point were previously reported in literature for other cross‐linked polymer systems, such as poly(vinyl alcohol) [47].

### 2.2 Comparison Between In Situ and Ex Situ UV Torsional Force Spectroscopy Measurements on PB Films

To verify the accuracy of the in situ UV torsional force spectroscopy mode, complementary ex situ measurements were performed. For this purpose, the PB film was partially covered with aluminum foil, illuminated using the same UV light source as for the in situ experiments (with the cantilever removed), and subsequently analyzed by torsional force spectroscopy at the transition between the foil‐covered and UV‐exposed regions. The corresponding procedure is visualized in Figure S10 of the Supporting Information.

**FIGURE 3:**
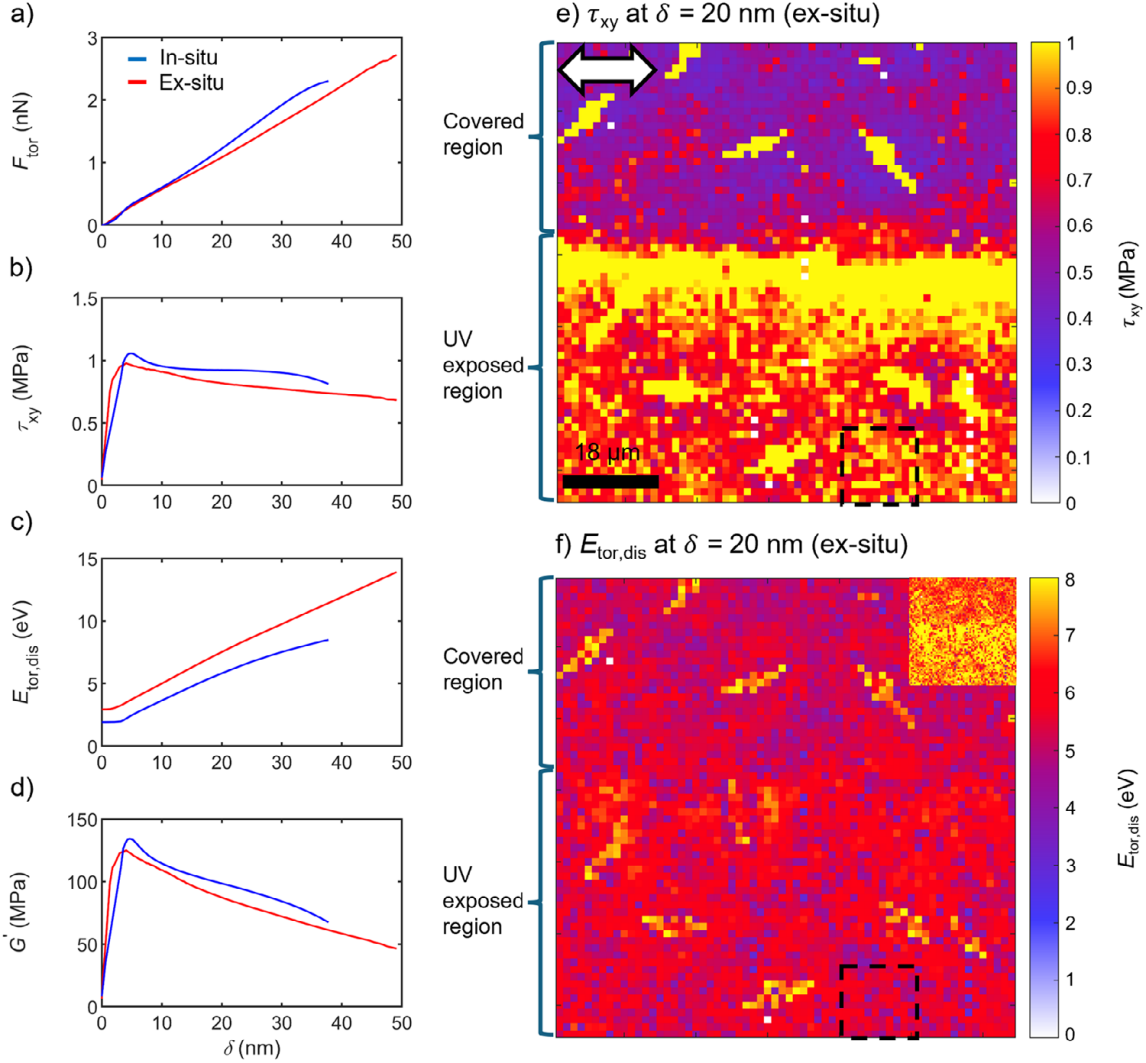
Comparison of mechanical quantities obtained from in situ (blue curves) and ex situ (red curves) UV torsional force spectroscopy: (a) torsional tip–sample force, (b) in‐plane shear stress, (c) torsional dissipated energy, and (d) storage shear modulus as functions of indentation depth. In situ data represent an average of 1024 measurement points over a 5 × 5 µm^2^ area (*n* = 1), while the ex situ data correspond to averages of 100 measurement points over a 14 × 14 µm^2^ area (*n* = 1). The measurement location for the ex situ data is indicated by black dashed frames in the depth profile images of (e) in‐plane shear stress and (f) torsional dissipated energy. The images show the quantities at an indentation depth of 20 nm. The white double arrow in (e) denotes the shearing direction of the AFM tip; the scale bar and arrow apply to both (e, f). The main image in panel (f) uses a wider color scale range to enhance defect visibility and to ensure that the area selected for averaging (black dashed frame) is not located within defect regions. The inset in (f) displays the same dissipated energy map with an adjusted color scale (0–6 eV) to better visualize the transition between the foil‐covered and UV‐exposed regions, resulting from partial coverage of the PB film surface with aluminum foil prior to UV illumination. All experiments were performed under otherwise identical conditions: UV illumination for 1640 s at an intensity of 0.72 mW cm^−2^, a vertical tip velocity of 2.5 µm s^−1^, a set point force of 5 nN, and a torsional oscillation amplitude of 0.75 nm.

Figure 3 compares the in situ (blue curves) and ex situ (red curves) measurements. Mechanical quantities as functions of tip indentation depth were derived using Equations ((1), (2), (3), (4), (5), (6), (7)) from the torsional force spectroscopy observables shown in Figure S11 (see Supporting Information). The in situ data represent averages of 1024 measurement points over a 5 × 5 µm^2^ area, whereas the ex situ data correspond to averages of 100 measurement points over a 14 × 14 µm^2^ area. The area where spectroscopy curves were chosen for analysis is indicated by black dashed frames in the depth profile image of in‐plane shear stress (see Figure 3e) and torsional dissipated energy (see Figure 3f). The main image of Figure 3f is displayed with a wider color scale range to enhance visibility of defect‐prone regions, in contrast to the corresponding inset, which uses a narrower scale to emphasize the transition between foil‐covered and uncovered regions.

The in situ and ex situ torsional tip–sample force curves (see Figure 3a) overlap well up to an indentation depth of 15 nm. Slight deviations appear at larger indentation depths (up to 35 nm), possibly due to UV light scattering at the edge of the aluminum foil that diminishes cross‐linking efficiency. Both in situ and ex situ in‐plane shear stress curves (see Figure 3b) exhibit a local maximum at approximately 5 nm indentation depth, which can be attributed to liquid meniscus formation at the tip–sample interface not accounted for in the contact area model [37]. The deviation between the in situ and ex situ in‐plane shear stress curves reaches up to 15 %, potentially caused by UV scattering effects, inaccuracies in preparing the PB solution with 4,4’‐bis(diethylamino)benzophenone (DEABP) and TRIS, or variations in the cantilever geometry of the same type.

Differences in the initial values of torsional dissipated energy (see Figure 3c) likely arise from day‐to‐day variations in temperature, humidity, or alignment of the detection and CleanDrive lasers, which affect the torsional eigenmode. Nevertheless, a good agreement is observed between the quantities measured by the in situ and ex situ approaches. The results demonstrate that the in situ configuration with a cantilever mounted in the atomic force microscope has a negligible effect on the cross‐linking of the PB films. The storage shear modulus curves (see Figure 3d) follow similar trends to the in‐plane shear stress curves since both quantities are closely related.

The depth profile image in Figure 3e and the inset in Figure 3f clearly show the distinct transition between the foil‐covered and UV‐exposed regions. Near the aluminum foil edge, the in‐plane shear stress increases (yellow color) to roughly 1 MPa compared to areas further away, consistent with UV light scattering effects at the edge. The foil‐covered region exhibits significantly lower in‐plane shear stress of approximately 0.5 MPa (blue color), while regions far from the transition show higher values (approximately 0.8 MPa, red color).

Yellow spike‐like spots appear in both the foil‐covered and UV‐exposed regions and indicate localized stiffness increases (see Figure 3e and f), possibly due to dewetting effects from interfacial reactions between the hydrophobic PB film and the underlying hydrophilic polyacrylamide (PAM) layer. The increase in in‐plane shear stress correlates with higher torsional dissipated energy in Figure 3f, consistent with enhanced irreversible polymer chain rearrangements induced by lateral tip motion in the UV‐exposed regions. The spatial and depth‐dependent evolution of these features can be seen in the corresponding in‐plane shear stress and dissipated energy maps provided as Movies S1 and S2 of the Supporting Information.

### 2.3 Influence of Polystyrene on the Cross‐Linking and In‐Plane Nanomechanical Properties of UV‐Illuminated PB Films

**FIGURE 4:**
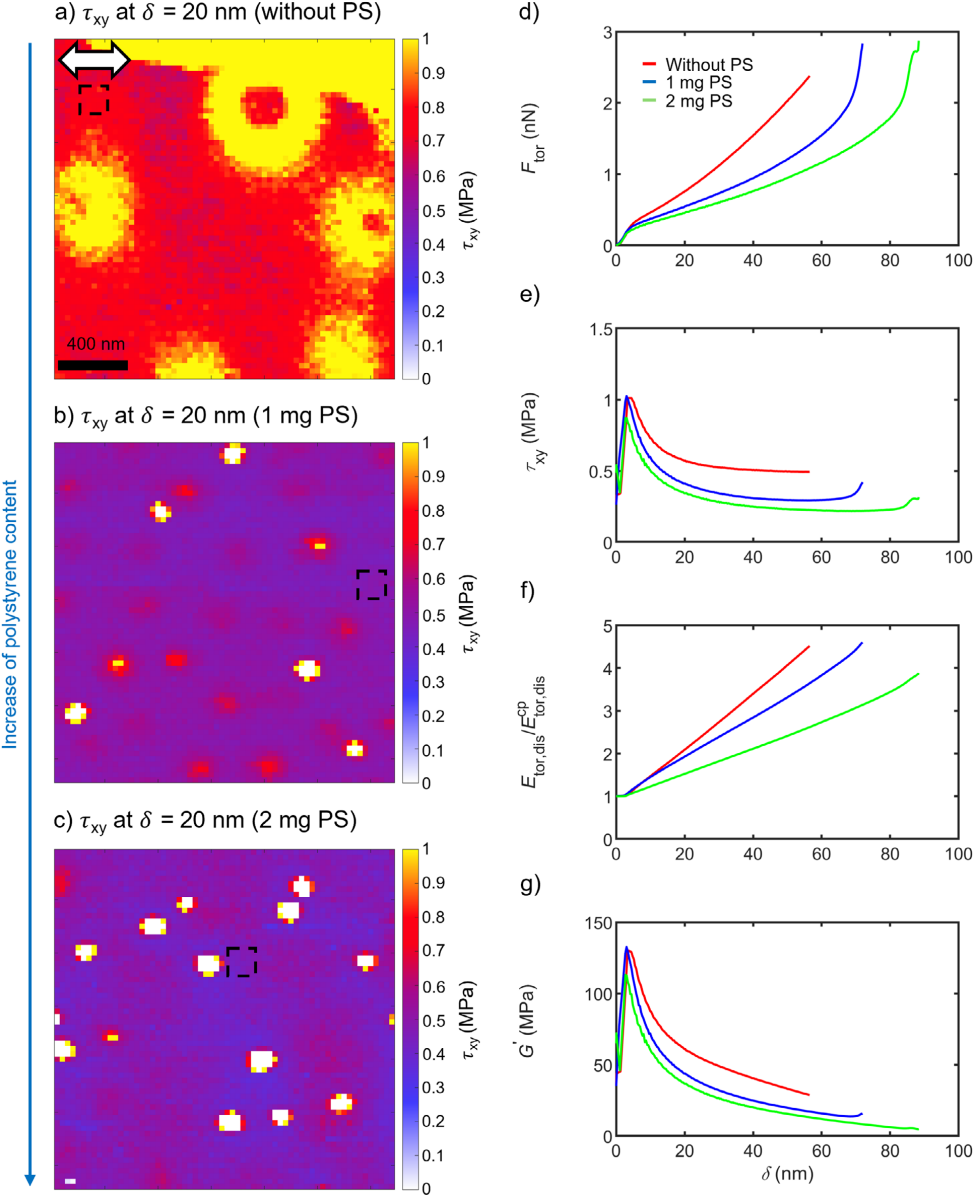
Depth profile images of in‐plane shear stress at 20 nm of indentation depth obtained from in situ UV torsional force spectroscopy on PB films containing identical amounts of DEABP and TRIS but varying contents of PS: (a) without PS, (b) 1 mg PS, and (c) 2 mg PS. The white spots in (b, c) mark the endpoints of the depth profile curves at the defined set point force of 5 nN, indicating the high stiffness of the PS domains at these locations. The white double arrow in (a) shows the shearing direction of the AFM tip. Both the scale bar and the double arrow shown in (a) also apply to (b, c). The dependence of the mechanical quantities on indentation depth is shown in panels (d–g): (d) torsional tip–sample force, (e) in‐plane shear stress, (f) torsional dissipated energy (normalized with respect to the dissipated energy at each contact point (*δ* = 0 nm)), and (g) storage shear modulus. Normalization of the torsional dissipated energy facilitates direct comparison between samples. The curves in (d–g) represent averages from 25 measurement points within a 156 × 156 nm^2^ area (*n* = 1), highlighted by black dashed frames in the depth profile images in (a–c). UV illumination was performed for 1640 s at an intensity of 0.72 mW cm^−2^. Torsional force spectroscopy mapping was conducted with a vertical tip velocity of 2.5 µm s^−1^, a set point force of 5 nN, and a torsional oscillation amplitude of 0.75 nm.

To investigate the effect of foreign‐component domains on the cross‐linking behavior of PB films, PS was added prior to spin coating. Depth profile images of in‐plane shear stress at an indentation depth of 20 nm for PB films containing constant amounts of DEABP and TRIS and with varying PS contents are shown in Figure 4.

The PB film without PS (see Figure 4a) exhibits a high in‐plane shear stress in the PB matrix, indicated by the red coloration. Yellow spots and rings in this image likely correspond to defects originating from small agglomerations of the photoinitiator, as reported in literature [40]. These defects differ from the white spots observed in films with 1 and 2 mg PS (see Figure 4b, c), which correspond to the endpoints of the depth profile curves due to the high stiffness at these locations. Comparison of the torsional force spectroscopy observables and mechanical quantities between the white spots in PB films with 1 and 2 mg PS and a pure PS film (see Figure S12 of the Supporting Information) reveals close agreement, indicating that the white spots correspond to phase‐separated PS domains. This interpretation is consistent with the droplet‐like morphology of phase‐separated PS domains as reported in the literature [48].

Increasing the PS content leads to a reduced overall in‐plane shear stress within the PB matrix, as evidenced by the more pronounced blue coloration in the 2 mg PS sample (see Figure 4c) relative to the 1 mg PS sample (see Figure 4b). Furthermore, larger and more numerous white spots in the 2 mg PS film provide additional evidence for the presence of phase‐separated PS domains. The evolution of these spatial features with indentation depth for PB films containing 0, 1, and 2 mg PS is visualized in Movies S3–S5 (Supporting Information).

To validate these observations, average curves of the torsional force spectroscopy observables were calculated from the regions marked by black dashed squares in Figure 4a–c. The corresponding mechanical quantities were derived using Equations ((1), (2), (3), (4), (5), (6)7) from the torsional force spectroscopy observables shown in Figure S13 (see Supporting Information).

The torsional tip–sample force curves (see Figure 4d) exhibit similar behavior up to an indentation depth of 5 nm, likely due to the formation of a liquid meniscus upon tip–sample contact. Beyond this point, the PB film without PS (red curve) exhibits a steeper increase and reaches its maximum indentation depth (≈55 nm) at a lower value than the films with 1 and 2 mg PS (≈70 nm and ≈90 nm). This suggests that the addition of PS influences the cross‐linking and stiffening behavior of the PB matrix, likely associated with the change in the spatial distribution of the cross‐linking agent TRIS.

To further test this hypothesis, X‐ray photoelectron spectroscopy (XPS) and Fourier transform infrared reflection absorption spectroscopy (FT‐IRRAS) measurements were performed (see Figures S14 and S15 of the Supporting Information). XPS revealed significant decreases in the peak intensity corresponding to the thiol and ester groups of the cross‐linking agent TRIS in PB films containing PS, which can be associated with surface‐sensitive redistribution effects, such as vertical phase separation or aggregation of TRIS at interfaces, when considering the limited probing depth of XPS of less than or equal to 10 nm with an Al *K_α_* source. To clarify which of these aforementioned scenarios most likely occurs, FT‐IRRAS measurements were conducted. A detailed discussion regarding possible spatial distributions of the TRIS cross‐linking agent molecules within the PB films upon addition of PS can be found in Section S14 of the Supporting Information. Based on this discussion, the surface arrangement of TRIS molecules is identified as the case with the highest probability to be present in the investigated PS‐containing PB films. This spatial rearrangement results in an ultrathin layer with thiol groups pointing toward the air interface, which screens the ester groups and causes a reduced peak intensity in the O1s signal of XPS. This represents a plausible explanation for the reduced stiffening detected upon UV illumination, without inducing a change in the intrinsic cross‐linking reaction mechanism.

The in‐plane shear stress curves (see Figure 4e) exhibit a local maximum at an indentation depth of approximately 5 nm, which can be attributed to the contact area model not accounting for liquid meniscus formation. Beyond 20 nm, the in‐plane shear stress remains constant up to the end of each curve. However, in the PB films with PS (blue and green curves), a slight increase in in‐plane shear stress curves appears near the end of the curves, likely due to the higher stiffness of the underlying PAM layer. In contrast, the PB film without PS does not show this increase, as its maximum indentation depth is too small to sense the PAM layer. Please note that the red curves in Figures 3b and 4e were recorded under identical sample preparation conditions (PB film without PS, UV intensity of 0.72 mW cm^−2^, and illumination time of 1640 s), but exhibit different decay behaviors after reaching their respective maximum in in‐plane shear stress. While the red curve in Figure 3b decays approximately linearly to slightly exponentially, the red curve in Figure 4e shows an exponential decrease. Such deviations typically stem from changes in tip geometry, which cannot be excluded in the present study.

The torsional dissipated energy curves were normalized with respect to the value at the respective contact point (*δ* = 0 nm) for better comparability due to the varying starting values of the torsional excitation amplitude (see Figure S13 of the Supporting Information). These normalized curves (see Figure 4f) reveal that the PB film without PS exhibits the steepest increase, likely due to a greater probability of irreversible rearrangements of polymer chains within the cross‐linked network during lateral tip motion when no phase‐separated PS domains are present.

The storage shear modulus (see Figure 4g) shows trends similar to the in‐plane shear stress, including the local maximum at 5 nm, but decreases continuously beyond this depth. A slight increase appears at ≈70 nm for the PS film with 1 mg PS, likely due to the mechanical influence of the PAM layer underneath.

Overall, the mechanical quantity curves are consistent with the trends observed in the depth profile images, demonstrating that in situ torsional force spectroscopy can serve as a predictive tool for optimizing additive concentrations and tailoring the mechanical properties of materials induced by UV illumination.

## 3 Conclusions

In this study, we applied in situ UV torsional force spectroscopy to investigate the temporal evolution of in‐plane nanomechanical properties in polymeric films during UV illumination. High‐resolution nanotomographic mapping combined with macroscopic swelling experiments enabled a direct correlation between local mechanical properties and macroscopic cross‐linking density.

Our results reveal that thiol‐ene cross‐linking under UV illumination induces a continuous increase in in‐plane shear stress, torsional dissipated energy, and storage shear modulus in PB films. The reliability of the in situ approach was confirmed by complementary ex situ measurements, with only minor deviations attributable to UV scattering effects and experimental variations.

To probe the sensitivity of the in situ method, we deliberately added PS to the PB films to form phase‐separated PS domains, serving as a model system for the investigation of the spatial heterogeneity. Supported by XPS and FT‐IRRAS analyses, torsional force spectroscopy consistently suggests that the observed reduced stiffness and altered energy dissipation stem from changes in the spatial distribution of the cross‐linking agent and the local mechanical properties of the PB matrix when the PS domains are present.

Overall, this work highlights in situ UV torsional force spectroscopy as a powerful tool for probing local mechanical properties and optimizing the additive concentrations to enhance material performance and service lifetime in polymeric applications. In addition, the method provides depth‐resolved insight into the evolution of the mechanical properties of PB films during UV illumination, allowing the detection of stiffness gradients and subsurface heterogeneity during curing. Beyond fundamental insights, this method offers a direct route to in situ process optimization, enabling precise adjustment of UV intensity, exposure time, and sequence of curing steps during polymer manufacturing. By allowing real‐time observation and quantification of mechanical evolution, this approach can significantly reduce experimental iterations and production costs in the development of advanced polymeric coatings and composites.

## 4 Experimental Section

### 4.1 Materials

Poly(1,4‐butadiene) (M_n_ = 32.2 kg mol^−1^, polydispersity index = 1.05) and polystyrene (M_n_ = 33 kg mol^−1^, polydispersity index = 1.07) were obtained from Polymer Source Inc. (Dorval, Canada). 4,4’‐bis(diethylamino)benzophenone (99 %) and trimethylolpropane tris(3‐mercaptopropionate) (95 %) were purchased from Sigma‐Aldrich Chemie GmbH (Taufkirchen, Germany) and used as photoinitiator and as cross‐linking agent, respectively. Polyacrylamide (M_n_ = 150 kg mol^−1^) was also obtained from Sigma‐Aldrich Chemie GmbH. Toluene (99.5 %) and n‐heptane (99 %) were supplied by Carl Roth GmbH + Co. KG (Karlsruhe, Germany). Ultrapure water (resistivity = 18.2 MΩ cm) was produced using a Direct‐Q 3 UV purification system (Millipore SAS, Molsheim, France). All chemicals were used as received without further purification.

### 4.2 Sample Preparation

A PAM solution (1 wt.%) was prepared by dissolving PAM (10 mg) in Milli‐Q water (1 mL) and stirring for 1 h at room temperature with a magnet.

The PB‐additive solution was prepared in two steps: first, poly(1,4‐butadiene) (9 mg) was dissolved in toluene (1 mL) under magnetic stirring for 2 h at room temperature; second, DEABP and TRIS (0.5 mg of each) were added to this solution under magnetic stirring for 1 h. For PS‐containing samples, 1–2 mg was added to the PB‐additive solution in a third step, followed by magnetic stirring for an additional hour.

Silicon (100) (Si/SiO_2_ substrates) wafers were purchased from LAM Research Corporation (Fremont, CA, USA) and cut into square pieces of 1.0–1.5 cm edge length. Dust particles and splinters from the cutting process were removed by blowing nitrogen gas across the wafer surface. To eliminate organic contaminants and render the wafer surface hydrophilic, the pieces were treated for 1 h with a UV/Ozone ProCleaner (BioForce Nanosciences Holdings, Inc., Virginia Beach, VA, USA). This treatment also ensured that the initial polyacrylamide coating spread uniformly across the wafer due to its low contact angle (<10°).

Spin coating was carried out using a WS‐400B‐6NPP/Lite spin coater (Laurell Technologies Corporation, Lansdale, PA, USA). For the PAM layer, the corresponding solution (100 µL) was dispensed onto the cleaned silicon substrate and spun for 30 s at 3000 rpm with an initial acceleration of 1044 s^−2^. The second layer was prepared by depositing the PB solution (100 µL) containing DEABP and TRIS on top of the PAM film and spinning for 60 s at 1000 rpm with an initial acceleration of 2088 s^−2^. For the PB samples containing PS, the same spin coating parameters were used.

### 4.3 Characterization of PB Films by In Situ and Ex Situ UV Torsional Force Spectroscopy

AFM experiments were performed with a Nanosurf DriveAFM atomic force microscope operated with Nanosurf Studio v11.8 software (Nanosurf AG, Liestal, Switzerland). Using the CleanDrive photothermal excitation setup (*λ* = 785 nm), the torsional eigenmode was excited by focusing the power‐modulated laser onto the cantilever backside slightly off its longitudinal symmetry axis [49]. For nanomechanical characterization of the PB films, PPP‐FMAuD cantilevers (NanoWorld AG, Neuchâtel, Switzerland) were used. These probes have a length *L*_c_ = 225 µm, a width *w*_c_ = 28 µm, a tip radius *R* < 10 nm, a nominal tip height *h*_tip_ = 15 µm, and a half‐cone angle *Θ* = 10°. The resonance frequency of the first flexural eigenmode in air was *f*_0,flex_ ≈ 60–70 kHz, and that of the first torsional eigenmode was *f*_0,tor_ ≈ 500–550 kHz. The flexural force constant *k*_flex_ could be determined to be 1.6–2 N m^−1^ using the thermal noise method [50], while Sader's method was employed to determine the torsional force constant *k*_tor_ [51], which was (1.5–2.0) × 10^−8^ N m rad^−1^, considering air with its density of *ρ*_f_ = 1.18 kg m^−3^ and viscosity of 1.86 × 10^−5^ kg m^−1^ s^−1^ as environment surrounding the tip. The in‐plane tip–sample force constant was derived from the torsional force constant divided by the squared tip height, giving *k*_ip_ of 65–90 N m^−1^ [52, 53]. The quality factor in air was *Q*_flex_ ≈ 180–200 for the flexural eigenmode and *Q*_tor_ ≈ 900–1020 for the torsional eigenmode. To calibrate cantilever deflection in the *z*‐direction (inverse vertical optical lever sensitivity, InvOLS), the AFM tip was pressed against a sapphire substrate to relate the *z*‐piezo actuator displacement (in nanometers) with the laser deflection signal on the photodiode (in volts). Lateral sensitivity was calibrated in contact mode by scanning with the fast scan axis perpendicular to the cantilever's long axis, while the slow scan axis was disabled. A normal force of ∼100 nN was applied over 10 nm scans on a silicon substrate, maintaining static friction. Under these conditions, the tip motion directly translates into lateral laser spot deflection on the photodiode. In this way, a lateral sensitivity of approximately 150 nm V^−1^ was determined. A phase‐locked loop (HF2PLL, Zurich Instruments, Zurich, Switzerland) was connected to the atomic force microscope to track the frequency shift of the first torsional eigenmode. Constant amplitude of this eigenmode was maintained by adjusting the excitation amplitude via the CleanDrive laser power, controlled by the built‐in proportional‐integral‐differential controller of the HF2PLL. Control of the laser was enabled by a Python script provided by Nanosurf AG. AFM torsional force spectroscopy was carried out in both in situ (under UV illumination) and ex situ (after UV illumination) modes to obtain depth profiles of the mechanical properties of the PB films. For the in situ measurements, the DriveAFM camera was removed, and a custom‐built UV light source was mounted on the atomic force microscope. The source consisted of Thorlabs GmbH (Bergkirchen, Germany) components: a fiber‐coupled LED (M365FP1, *λ* = 365 nm), fiber patch cable (M131L02), fiber collimation package (F240FC‐A, numerical aperture = 0.51), T‐Cube power supply (LEDD1B), and power cable (KPS201). The collimator was mounted in a custom 3D‐printed holder, providing stable alignment during illumination. The PB sample on the stage was illuminated at normal incidence (90°) by UV light directed through the AFM scan head and cantilever holder. Intensity was calibrated with an MVL‐D‐0111‐3 optometer (SUSS MicroTec SE, Garching, Germany) placed beneath the cantilever holder and exposed to UV light while varying the T‐Cube power supply current (calibration data in Figure S5, Supporting Information). The UV light intensity was varied by adjusting the applied current to generate master curves for the in‐plane nanomechanical properties. For the ex situ measurements, a portion of the PB sample was covered with aluminum foil to create a sharp edge. The cantilever holder was positioned at the sample surface without a cantilever. Subsequently, the UV source was mounted on the atomic force microscope, and UV illumination was carried out under otherwise identical conditions. This procedure, illustrated in Figure S10 of the Supporting Information, produced a distinct transition between the UV‐exposed region and the foil‐covered region.

In situ UV torsional force spectroscopy was performed on PB samples without PS using nine force maps, each covering an area of 5 × 5 µm^2^ with 32 × 32 measurement points, a vertical tip velocity of 2.5 µm s^−1^, a torsional oscillation amplitude of 0.75 nm, and a set point force of 5 nN, within the maximum 100 × 100 µm^2^ scan range of the DriveAFM. Each force map required 410 s to acquire.

To compare the UV‐induced cross‐linking behavior of PB films with and without PS, in situ UV torsional force spectroscopy was carried out on each sample by acquiring a single force map covering an area of 2 × 2 µm^2^ with 64 × 64 measurement points, a vertical tip velocity of 2.5 µm s^−1^, a torsional oscillation amplitude of 0.75 nm, and a set point force of 5 nN. For these measurements, the UV illumination time was fixed at approximately 1640 s with an intensity of 0.72 mW cm^−2^.

Ex situ torsional force spectroscopy was performed at the interface between foil‐covered and uncovered regions. A 90 × 90 µm^2^ force map (64 × 64 points) was recorded with a tip velocity of 2.5 µm s^−1^, a torsional amplitude of 0.75 nm, and a set point force of 5 nN.

During approach and retraction of the AFM tip, the vertical static force, torsional frequency shift, and torsional excitation amplitude were recorded as functions of *z*‐sensor position. The indentation depth *δ* of the tip into PB films was calculated from the relationship *δ* = *z* – *d*, where *z* represents the *z*‐sensor position and *d* the vertical cantilever deflection at that position.

### 4.4 AFM Data Processing and Analysis

The acquired force maps were processed based on custom Matlab scripts (MATLAB R2024a, MathWorks Inc., Natick, MA, USA) following procedures similar to those reported in literature [36, 37].

### 4.5 Macroscopic Swelling Experiments

After spin coating, PB films without PS were illuminated with UV light at *λ* = 365 nm using a mask aligner (MJB4, SUSS MicroTec SE, Garching, Germany), as the chosen photoinitiator exhibited strong absorption at this wavelength. Illumination was performed in full‐field exposure mode (without a mask), and the intensity (16 mW cm^−2^) was monitored with an optometer (MVL‐D‐0111‐3, SUSS MicroTec SE, Garching, Germany). The UV exposure duration was varied between 0 and 600 s.

The macroscopic cross‐linking density of the UV‐illuminated PB samples was determined by monitoring the swelling behavior of the films floating on water, following the method of Saito et al [38]. To standardize film size, adhesive tape with a 1 cm × 1 cm square cutout was placed on the silicon wafer before spin coating. After coating, the tape was removed, leaving patterned PB films for illumination (see Figure S6 of the Supporting Information). A water‐soluble PAM layer was required to release the PB films from the silicon substrates upon immersion in Milli‐Q water. Floating films were imaged before and after swelling in n‐heptane. For this purpose, Milli‐Q water (15 mL) was poured into a glass petri dish. After the detachment of the PB films from the silicon substrates, n‐heptane (5 mL) was carefully added to avoid film rupture. The sizes of the unswollen and swollen PB films were determined from optical images using the ImageJ v1.53t software (College Park, MD, USA). The Flory–Rehner equation was applied for deriving the macroscopic cross‐linking density of the UV cross‐linked PB films [10].

### 4.6 X‐Ray Photoelectron Spectroscopy

To investigate the effect of phase‐separated PS domains on the surface chemistry of the PB films, XPS was carried out on pure PB as well as PB films containing DEABP and TRIS, with and without PS. The measurements were performed using an ESCALab‐250 instrument (Thermo Fisher Scientific, East Grinstead, United Kingdom). For these experiments, a monochromatic Al *K_α_* X‐ray source (*hν* = 1486.6 eV) with a spot size of 650 µm and an output power of 200 W was used. The XPS measurement chamber has a vacuum level better than 5 × 10^−10^ mbar. High‐resolution core‐level spectra, as well as survey spectra, were obtained using pass energies of 10 and 50 eV, respectively. Since the PB films on silicon wafers exhibited only a surface charging between +1.2 and +1.7 eV, charge compensation by electron flooding was not used in order to avoid any chemical modifications to the polymeric films. The surface charging was corrected by shifting all spectra so that the main C1s (C sp^3^) peak matched 285.0 eV for each sample. Prior to use, the XPS apparatus was calibrated using a linearity test with Cu2p_3/2_, Ag3d_5/2_, and Au4f_7/2_ core levels and the Ag Fermi edge.

### 4.7 Fourier Transform Infrared Reflection Absorption Spectroscopy

FT‐IRRAS was performed on the PB films using a VERTEX 80v Fourier transform infrared spectrometer (Bruker Optics GmbH & Co. KG, Ettlingen, Germany). A liquid N_2_‐cooled MCT detector (D315/B) was used, combined with an 8° grazing angle specular reflection accessory (SPECAC P/N GS19650) equipped with a 1.5 mm aperture, resulting in an elliptical IR beam spot on the sample with dimensions of 1.5 mm × 10.8 mm. Both the sample and the background spectra were recorded with 1000 scans in an evacuated environment with a spectral resolution of 4 cm^−1^.

### 4.8 Statistical Analysis

The statistical analysis was performed in this study with *n* denoting the number of independent samples. For each data point of the master curves shown in Figure 2a, b, the average and standard deviations were taken from 1024 points of a single force map at the respective UV light intensity and illumination time (*n* = 1). This data is presented as mean ± SD. The depth profile curves illustrated in Figure 3a–d were determined by averaging over 1024 points for the in situ data (*n* = 1) and over 100 points for the ex situ data (*n* = 1). The curves shown in Figure 4d–g were determined by taking the average of 25 measurement points for each investigated PS content (*n* = 1). For Figure 4f, the torsional dissipated energy was normalized with respect to the dissipated energy at every contact point (*δ* = 0 nm). MATLAB R2024a (MathWorks Inc., Natick, MA, USA) was used for calculating the average and standard deviations of the data points and error bars, as well as for plotting the graphs in all figures of the study.

## Funding

Deutsche Forschungsgemeinschaft (DFG, German Research Foundation) (Grant number: 467382377)

## Conflicts of Interest

The authors declare no conflicts of interest.

## Supporting information




**Supporting File 1**: smtd70548‐sup‐0001‐SuppMat.pdf.

**Supporting File 2**: smtd70548‐sup‐0002‐MovieS1.mov.

**Supporting File 3**: smtd70548‐sup‐0003‐MovieS2.mov.

**Supporting File 4**: smtd70548‐sup‐0004‐MovieS3.mov.

**Supporting File 5**: smtd70548‐sup‐0005‐MovieS4.mov.

**Supporting File 6**: smtd70548‐sup‐0006‐MovieS5.mov.

## Data Availability

The data that support the findings of this study are available from the corresponding author upon reasonable request.
