# Supplementary material for: In Situ UV Torsional Force Spectroscopy for Real‐Time Mapping of Cross‐linking Kinetics and Mechanical Properties in Polymeric Films
Source: Small Methods. 2026 Mar 22;10(8):e02210. doi: 10.1002/smtd.202502210 (PMC13103639; doi:10.1002/smtd.202502210)
Supplement: Supplementary file 1 — Supporting File 1: smtd70548‐sup‐0001‐SuppMat.pdf. [file SMTD-10-e02210-s005.pdf]

WILEY-VCH

Supporting Information

In situ UV Torsional Force Spectroscopy for Real-Time Mapping of Cross-linking Kinetics and Mechanical Properties in Polymeric Films

*Marvin Hoffer**, *Felix Petersein*, *Martin Dehnert*, *Tobias Andreas Lintner*, *Hikmet Sezen*, *Jan Philipp Hofmann*, *and Christian Dietz**

Movies

Movie S1. In-plane shear stress maps of ex situ torsional force spectroscopy on PB film without PS at different AFM tip indentation depths.

Movie S2. Torsional dissipated energy maps of ex situ torsional force spectroscopy on PB film without PS at different AFM tip indentation depths.

Movie S3. In-plane shear stress maps of in situ UV torsional force spectroscopy on PB film without PS at different AFM tip indentation depths.

Movie S4. In-plane shear stress maps of in situ UV torsional force spectroscopy on PB film with 1 mg PS at different AFM tip indentation depths.

Movie S5. In-plane shear stress maps of in situ UV torsional force spectroscopy on PB film with 2 mg PS at different AFM tip indentation depths.

S1. Thiol-ene Reaction Mechanism

To understand the evolution of the in-plane nanomechanical properties of ultraviolet (UV)-illuminated poly(1,4-butadiene) (PB) films, the thiol-ene reaction mechanism induced by UV illumination is illustrated in **Figure S1**.[1] Upon exposure to UV light at 365 nm, the photoinitiator 4,4'-bis(diethylamino)benzophenone (DEABP) is excited into two radical states (highlighted in red in Figure S1a). These radicals abstract hydrogen atoms from the thiol groups (–SH) of trimethylolpropane tris(3-mercaptopropionate) (TRIS) cross-linking agent molecules, as indicated by the light green arrows in Figure S1a. The resulting TRIS radicals then attack the double bonds of PB homopolymer chains (see brown arrows in Figure S1a), leading to covalent bond formation between PB and TRIS, i.e., cross-linking (see pink circles in Figure S1a). During this process, a radical is generated at the neighboring carbon atom of the PB chain. The reaction can either terminate when a hydrogen atom attaches to this radical, as shown by the dark green arrow in Figure S1a, or propagate as a chain-reaction through further bond formation between PB and TRIS molecules (see orange circles and arrows in Figure S1a). This sequence results in the formation of a cross-linked network, schematically represented by the black and blue structures in Figure S1b.

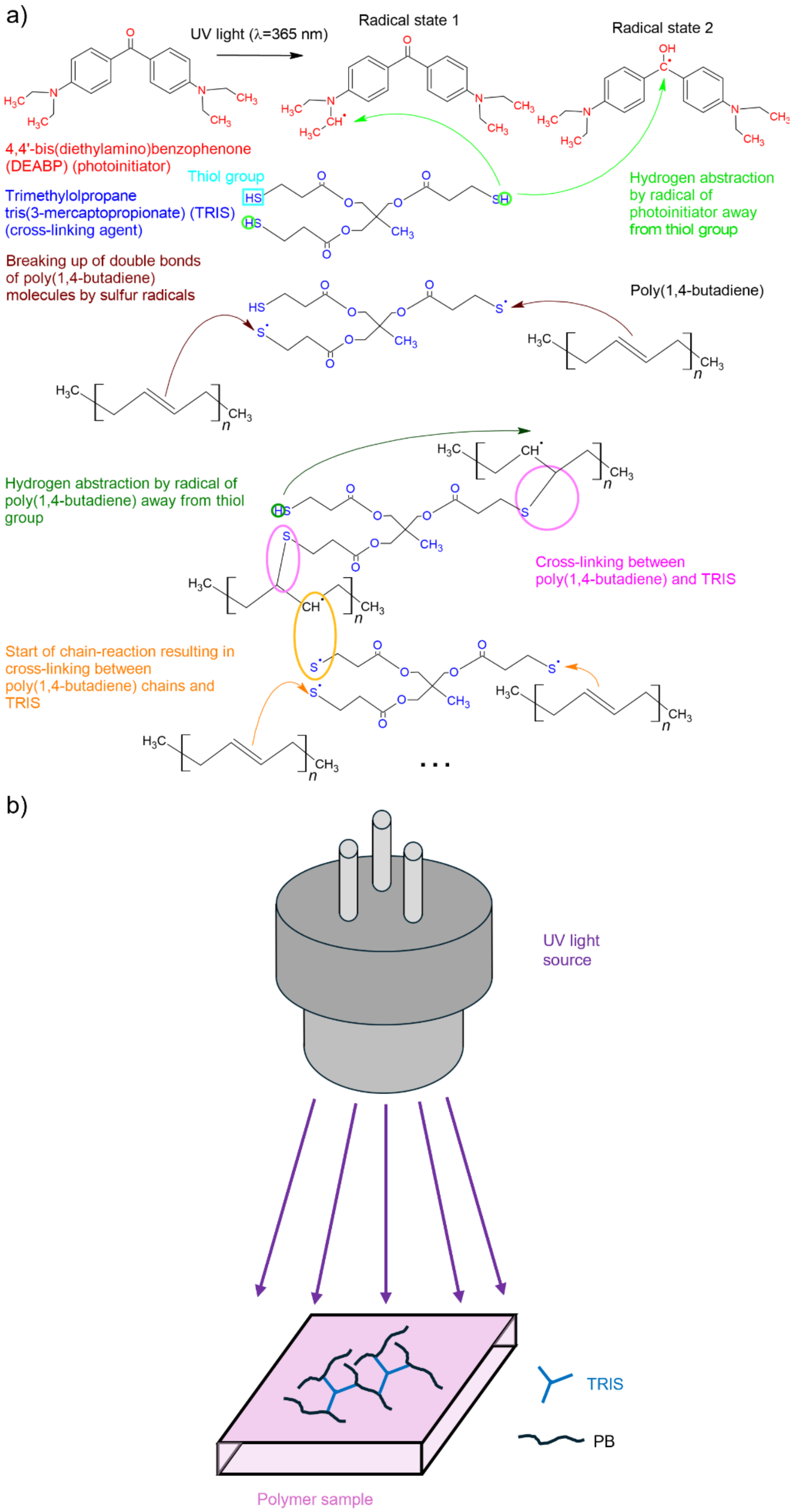

Figure S1: Schematic illustration of a) the UV-induced cross-linking reactions and b) polymer network formation under UV illumination at a wavelength of 365 nm.

S2. Setup for In situ UV Torsional Force Spectroscopy

The experimental setup to perform in situ UV torsional force spectroscopy is shown in **Figure S2**. The sample stage of the atomic force microscope is mounted on top of an inverted optical microscope, which itself is positioned on an air-balanced platform to minimize vibrations during torsional force spectroscopy measurements inside a noise-canceling chamber. The chamber windows are covered with aluminum foil to protect the user from UV illumination during operation. The DriveAFM is placed on the sample stage, and the camera is removed before the measurements to mount the fiber collimator of the UV light source into a 3D-printed holder on top of the atomic force microscope. The fiber collimator is connected via a fiber patch cable to the fiber-coupled LED, which is powered by a controlled current supply.

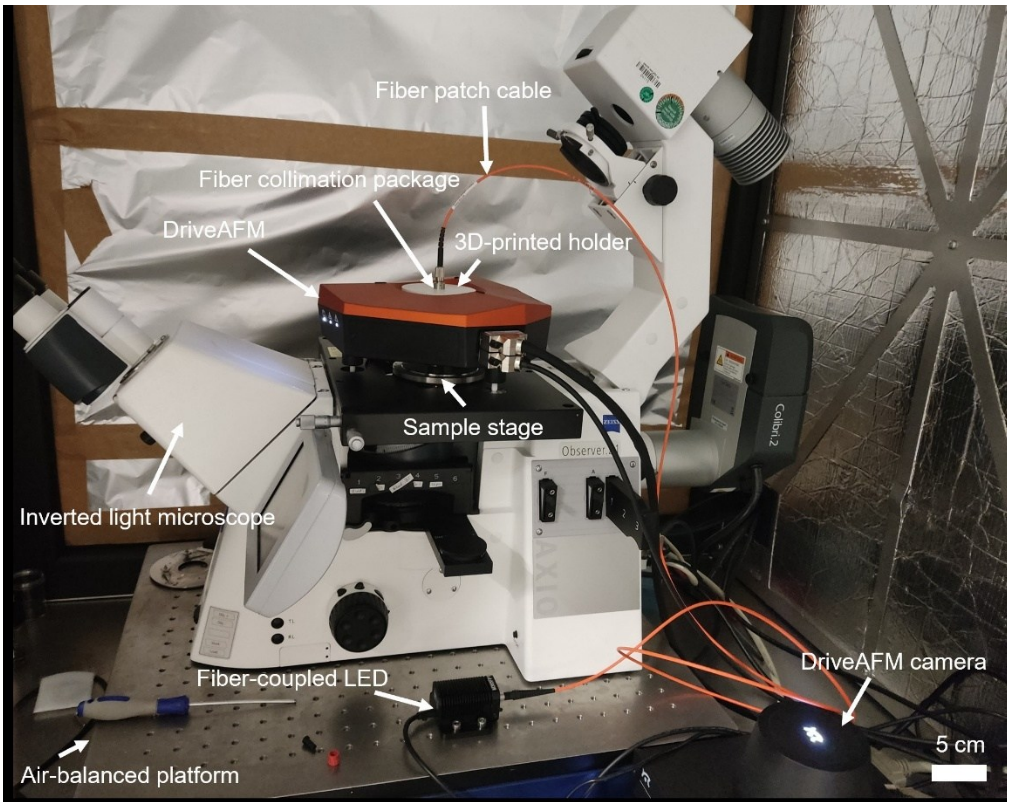

Figure S2: Experimental setup for performing in situ UV torsional force spectroscopy with the DriveAFM.

S3. Step-by-step Guides for Linking Torsional Force Spectroscopy Raw Data to Absolute In-plane Nanomechanical Properties

To link the raw data from torsional force spectroscopy to the quantitative in-plane nanomechanical properties, step-by-step guides are provided in **Figure S3** and **Figure S4** in this section of the Supporting Information.

The conversion of the torsional frequency shift Δf_{tor} as a function of z-sensor position into the storage shear modulus G' as a function of indentation depth δ of the AFM tip is shown in Figure S3. In the first step, the z-sensor position (see Figure S3a) is converted into the indentation depth δ (see Figure S3b) by subtracting the vertical cantilever deflection d from the z-sensor position z. Afterward, the torsional tip–sample force F_{tor} is determined from the torsional frequency shift using Equation (1) from the main text (see Figure S3c). For this purpose, the in-plane tip–sample force constant k_{ip} is required, which can be determined by dividing the torsional force constant k_{tor} by the squared tip height h_{tip}. For the torsional force constant, Sader's method is applied,[2] considering the geometrical measures of the used cantilever (length L_{c} and width w_{c}), the properties of the air environment (density ρ_{f} and viscosity η_{f} for the imaginary part of the hydrodynamic function $\Gamma_{\mathrm{i}}^{\mathrm{t}}$), the resonance frequency $f_{0,\mathrm{tor}}$ of the torsional eigenmode, and the quality factor Q_{tor} of the torsional eigenmode as quotient of resonance frequency and full width at half maximum (FWHM) of the resonance peak (see red curve in Figure S3b). The torsional oscillation amplitude A_{tor} needs to be converted from the unit of volts into nanometers. Therefore, the oscillation amplitude in volts is multiplied with the lateral sensitivity s_{lat} derived as inverse slope from the lateral laser spot deflection on the photodiode and the relative scan position (see purple dashed lines in Figure S3c). The torsional tip–sample force is further transformed, as shown in Figure S3d, into the in-plane shear stress by dividing according to Equation (2) of the main text by the tip–

sample contact area A_c. The shell surface of a sphere is considered for the tip–sample contact area if the indentation depth is smaller or equal to the tip radius R (see Equation (3) of the main text). When the indentation depth exceeds the tip radius, the tip–sample contact area represents the sum of the shell surface of the sphere and the conical sidewalls of the tip (see Equation (4) of the main text). Please refer to our recent study for the derivation of the equations for calculation of the tip–sample contact area.[3] In the last step (see Figure S3e), the in-plane shear stress is converted into the storage shear modulus by multiplying the in-plane shear stress with the difference between polymer film thickness h and indentation depth as well as by dividing by the torsional oscillation amplitude A_{tor} (see Equation (6) of the main text).

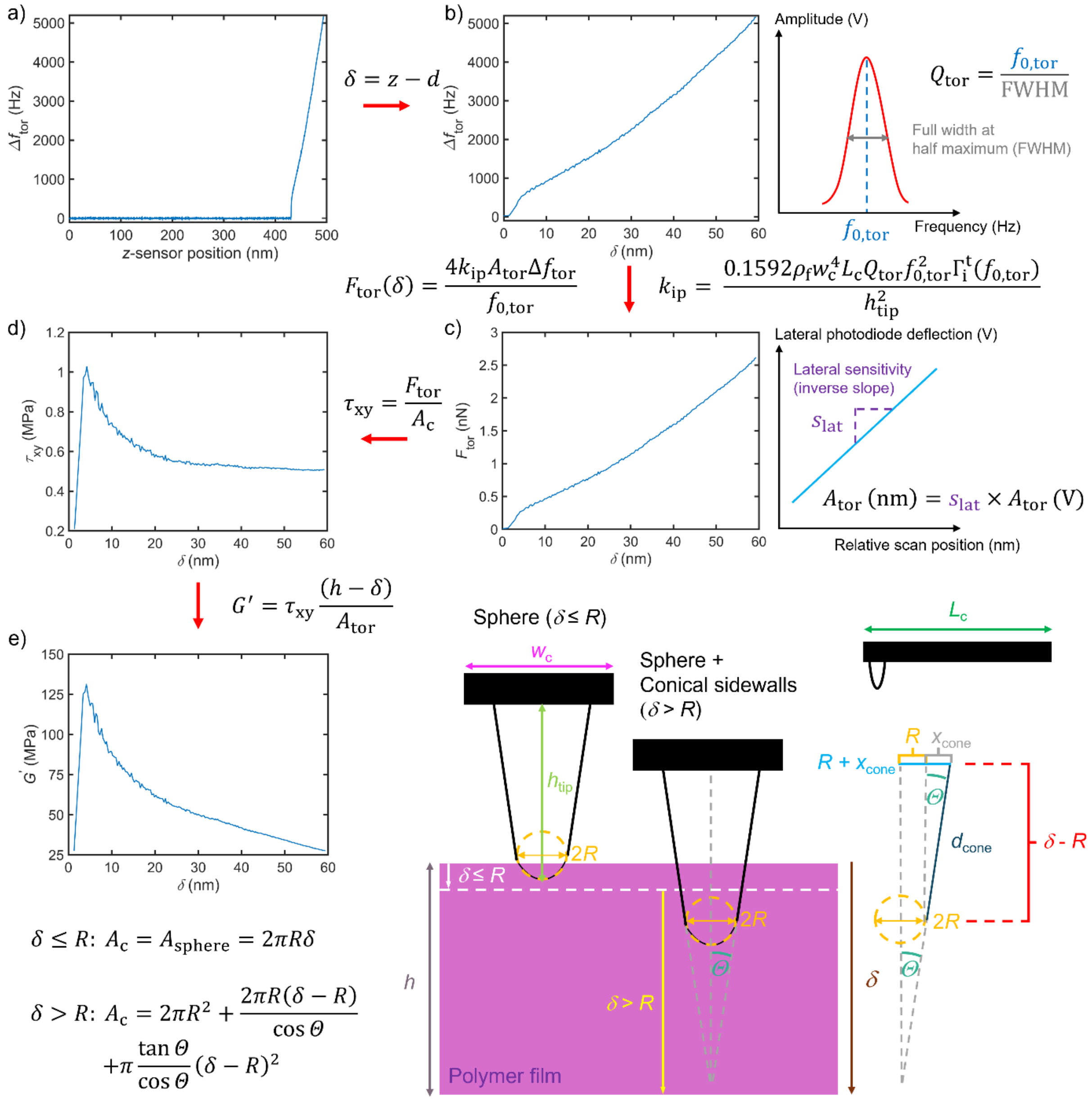

Figure S3: Step-by-step guide from conservative torsional force spectroscopy raw data to absolute in-plane nanomechanical properties: From torsional frequency shift Δf_{tor} to storage shear modulus G'. Conversion of a) z-sensor position z into b) indentation depth δ as well as torsional frequency shift Δf_{tor} into c) torsional tip–sample force F_{tor}, d) in-plane shear stress τ_{xy}, and e) storage shear modulus G'. In this figure, d denotes the vertical cantilever deflection, Q_{tor} the quality factor of the torsional eigenmode, $f_{0,tor}$ the corresponding resonance frequency of the torsional eigenmode, FWHM the full width at half maximum of the resonance peak, ρ_f the density of the air environment, w_c the width and L_c the length of the used cantilever, Γ_i^t the imaginary part of the hydrodynamic function, h_{tip} the tip height, k_{ip} the in-plane tip–sample force

constant, A_{tor} the torsional oscillation amplitude, s_{lat} the lateral sensitivity, A_c the tip–sample contact area, h the polymer film thickness, R the tip radius, Θ the half-cone angle of the tip, d_{cone} the length of the conical sidewalls of the tip, and x_{cone} the length difference between conical sidewalls and tip radius.

The conversion of the torsional free amplitude A_0 as a function of z-sensor position into the torsional dissipated energy $E_{tor,dis}$ as a function of indentation depth δ of the AFM tip is shown in Figure S4. The z-sensor position (see Figure S4a) is converted into the indentation depth δ (see Figure S4b) by subtracting the vertical cantilever deflection d from the z-sensor position z. The torsional free amplitude A_0 is transformed into the torsional excitation amplitude a_{tor} by dividing by the quality factor Q_{tor} of the torsional eigenmode (see Figure S4c). The torsional excitation amplitude needs to be converted from the unit of volts to nanometers by multiplying with the lateral sensitivity s_{lat} (see Figure 4d). In the last step, the torsional excitation amplitude is converted into the torsional dissipated energy using Equation (7) of the main text (see Figure S4e).

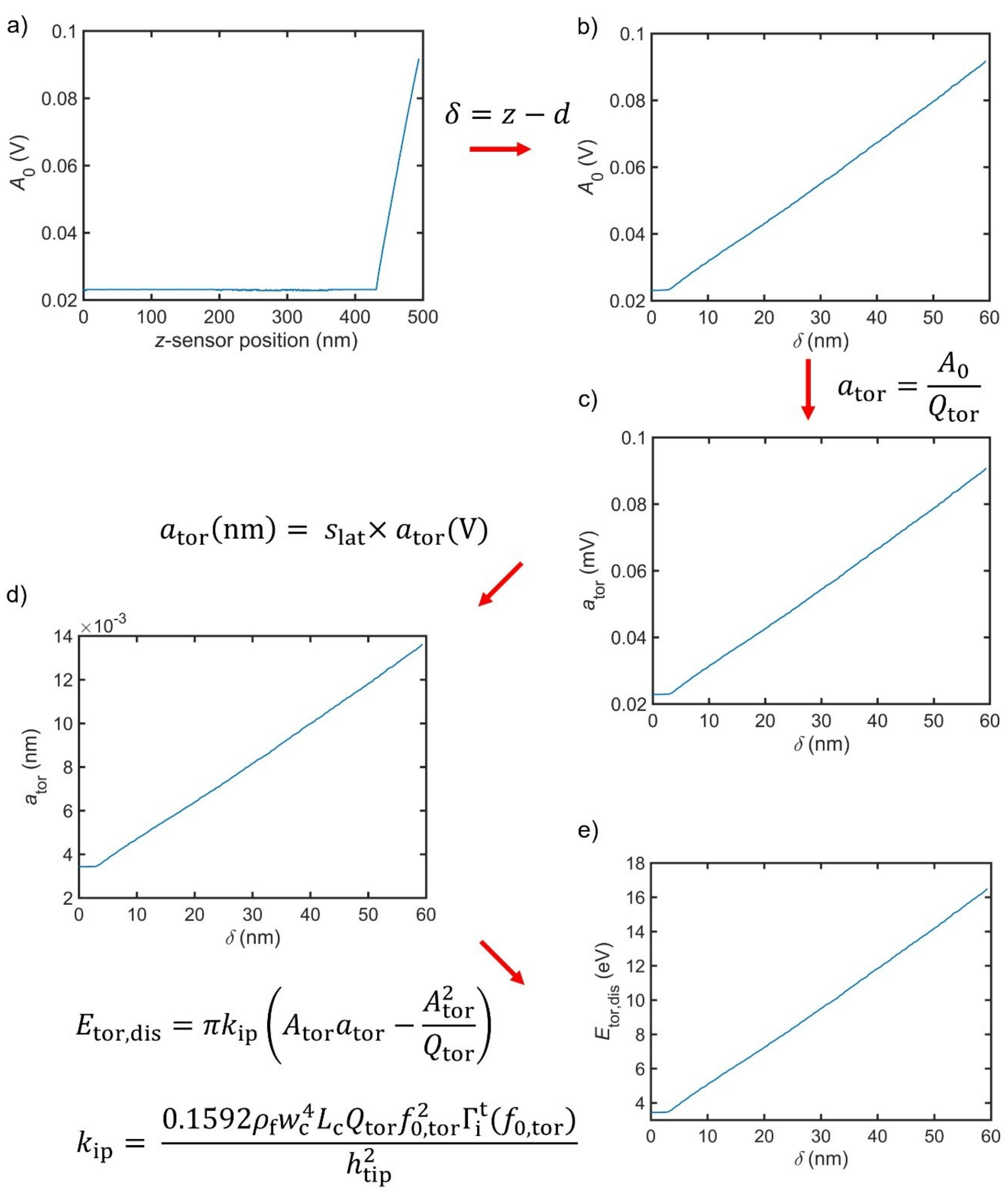

Figure S4: Step-by-step guide from dissipative torsional force spectroscopy raw data to absolute in-plane nanomechanical properties: From torsional free amplitude A_0 to torsional dissipated energy $E_{\mathrm{tor,dis}}$. Conversion of a) *z*-sensor position *z* into b) indentation depth δ as well as torsional free amplitude into torsional excitation amplitude a_{tor} in the unit of c) volts and d) nanometers, and e) torsional dissipated energy. In this figure, *d* denotes the vertical cantilever deflection, Q_{tor} the quality factor of the torsional eigenmode, s_{lat} the lateral sensitivity, ρ_{f} the

density of the air environment, w_c the width and L_c the length of the used cantilever, $f_{0,tor}$ the resonance frequency of the torsional eigenmode, Γ_i^t the imaginary part of the hydrodynamic function, h_{tip} the tip height, k_{ip} the in-plane tip–sample force constant, and A_{tor} the torsional oscillation amplitude.

S4. Calibration of UV Light Intensity

To derive the deposited dose of UV light during in situ UV torsional force spectroscopy, calibration of the UV light intensity was performed. The measurement probe of an optometer was positioned beneath the DriveAFM with the cantilever holder mounted. The current supplied to the UV light source was then incrementally adjusted using a power supply. The resulting calibration curve, depicted in **Figure S5**, demonstrates the increase in intensity with increasing current.

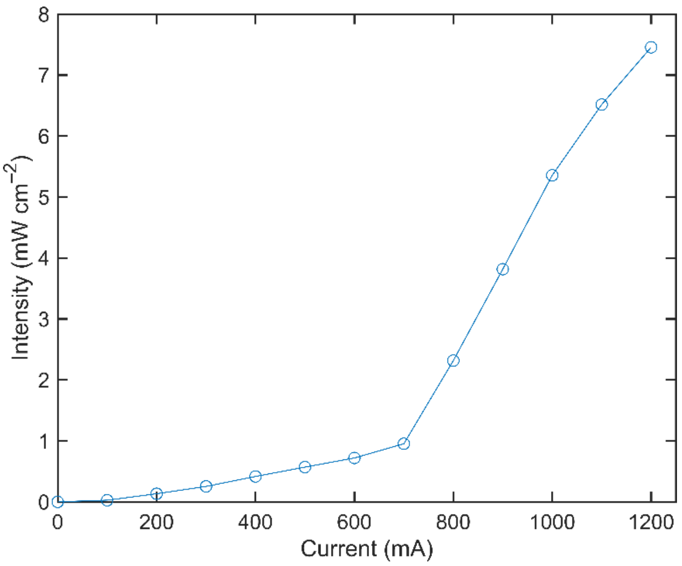

Figure S5: Calibration curve of UV light source used for in situ UV torsional force spectroscopy.

S5. Patterning Procedure of PB Films for Macroscopic Swelling

To ensure accurate determination of the cross-linking density, PB films were patterned to maintain identical spatial dimensions and consistent starting conditions for swelling. The procedure is depicted in **Figure S6**. First, the silicon substrate was treated with UV/ozone for one hour to create a hydrophilic surface, promoting uniform spreading of the polyacrylamide (PAM) solution (see Figure S6a). Next, spin coating was carried out on the silicon substrate with attached adhesive tape with 1 cm × 1 cm square cutout: the PAM solution was deposited as the first layer, followed by the PB solution containing the photoinitiator DEABP and the cross-linking agent TRIS as the second layer, as shown in Figure S6b. After coating, the adhesive tape was removed, and the patterned PB films were exposed to UV light using a mask aligner (see Figure S6c).

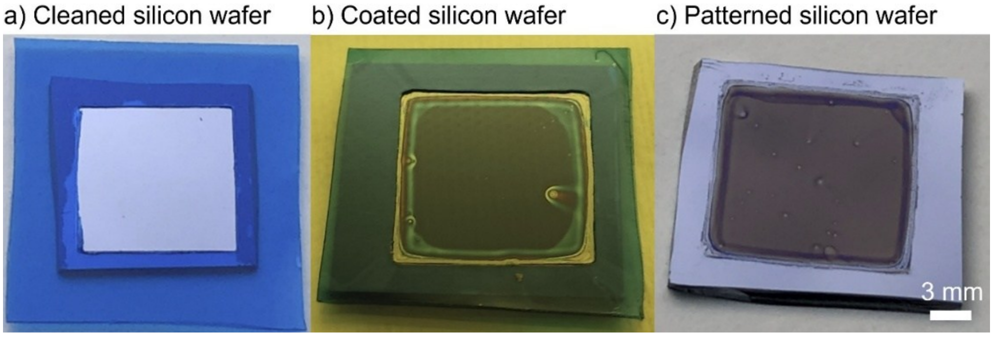

Figure S6: Visual sequence of the patterning procedure for PB films: a) silicon substrate with attached adhesive tape after UV/ozone treatment, b) coated silicon substrate with adhesive tape after spin coating, and c) patterned PB film on silicon substrate before UV illumination.

S6. Fitting of Macroscopic Cross-Linking Density as a Function of UV dose

To correlate the in-plane nanomechanical properties determined by torsional force spectroscopy with the cross-linking density from macroscopic swelling of the UV-illuminated PB films, the

UV illumination time was converted into UV dose by multiplying the exposure time with the UV light intensity of the mask aligner (16 mW cm^{-2}). The macroscopic cross-linking density as a function of the resulting UV dose is depicted in **Figure S7**. The shown data points (blue) were fitted using a dual saturation function (red curve). This choice for fitting can be attributed to the occurrence of two distinct regimes of cross-linking, which exhibit different reaction rates. In the first regime (see yellow boxes in Figure S7), cross-linking primarily occurs at the surface based on the inner shielding effect: [4], [5] UV light of the highest intensity is absorbed at the surface of the PB film, resulting in a gradient in cross-linking and stiffness within the PB film. Therefore, the first regime represents surface network formation, since the bulk of the film is partially unreacted due to shielding by the cross-linked surface (see **Figure 2**f of the main text). The reaction rate within this regime is reaction-limited, depending on the local concentration of the photoinitiator.

In the second regime (see orange boxes in Figure S7), cross-linking expands further into the bulk of the PB film (bulk network formation). This regime is diffusion-limited due to steric hindrance of the cross-linking agent. [6]

Overall, the dual saturation function considers the combination of both regimes representing fast cross-linking at the surface and slower cross-linking in the bulk and leads to a physically reasonable and excellent fit regarding the experimental data from the swelling experiments.

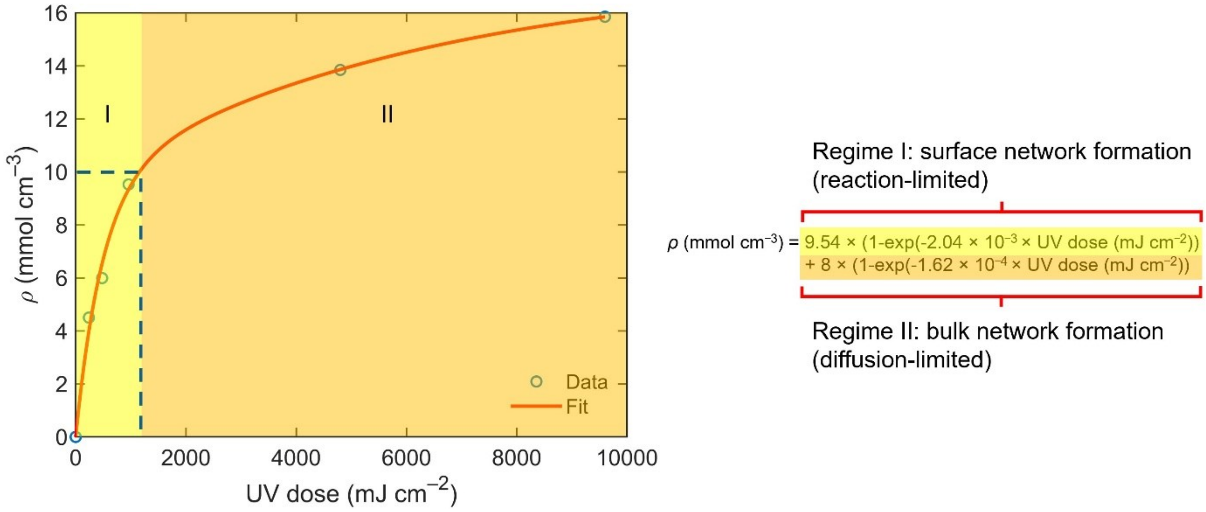

Figure S7: Macroscopic cross-linking density as a function of UV dose (blue hollow points) with dual saturation fit (red). The resulting equation for the fit is displayed next to the graph. The yellow and orange boxes in the graph in combination with the Romanian numbers indicate the different regimes of cross-linking. The light blue dashed lines highlight the transition point between the two regimes of cross-linking.

S7. Torsional Force Spectroscopy Observables and Mechanical Quantities as Functions of Indentation Depth for Varied UV Illumination Times and Intensities

To create master curves for the in-plane nanomechanical properties of the UV-illuminated PB films, both the UV illumination time and the UV light intensity were systematically varied during in situ UV torsional force spectroscopy. The resulting observables—vertical static force F_{stat}, torsional frequency shift Δf_{tor}, and excitation amplitude a_{tor}—are shown as functions of the AFM tip indentation depth and UV illumination time at different intensities in **Figure S8**. Each curve in these graphs represent the average of 1024 measurement points of a single force map recorded at the defined UV illumination time.

To assess the intrinsic heterogeneity of the spin coated PB films, torsional force spectroscopy was first performed without UV illumination (0 mW cm^{-2}), as shown in Figure S8a. All vertical static force curves (see Figure S8a) propagate in the attractive regime up to an indentation depth of 70 nm, reflecting the formation of the liquid meniscus upon first tip–sample contact and the stickiness of the PB films in the absence of UV-induced cross-linking.[7] Beyond this depth, a steep increase into the repulsive regime appears until the defined set point force of 5 nN is reached, which is attributed to the higher stiffness of the underlying PAM layer. Only minor deviations between vertical static force curves are observed in this regime, indicating negligible heterogeneity across the PB films for in situ torsional force spectroscopy.

Similar trends are observed in the torsional frequency shift and excitation amplitude. A clear change in the slope of the torsional frequency shift coincides with the transition from attractive to repulsive behavior in the vertical static force at approximately 70 nm of indentation depth.

Upon UV illumination (see Figure S8b–d), the maximum indentation depth reached at the same set point force of 5 nN systematically decreases with longer illumination times. This trend appears in both out-of-plane (vertical static force) and in-plane observables (torsional frequency shift and excitation amplitude), indicating UV-induced stiffening of the PB films in both directions. The stiffening is consistent with the thiol-ene reaction mechanism shown in Figure S1, where UV-induced cross-linking between PB chains and TRIS cross-linking agent molecules generates a three-dimensional polymer network.

The transition from attractive to repulsive regimes occurs in the vertical static force curves at progressively smaller indentation depths with increasing UV illumination time, further supporting this stiffening behavior. Moreover, higher UV light intensities lead to a steeper rise

in all three torsional force spectroscopy observables, suggesting a faster reaction rate of the cross-linking process and accelerated stiffening kinetics.

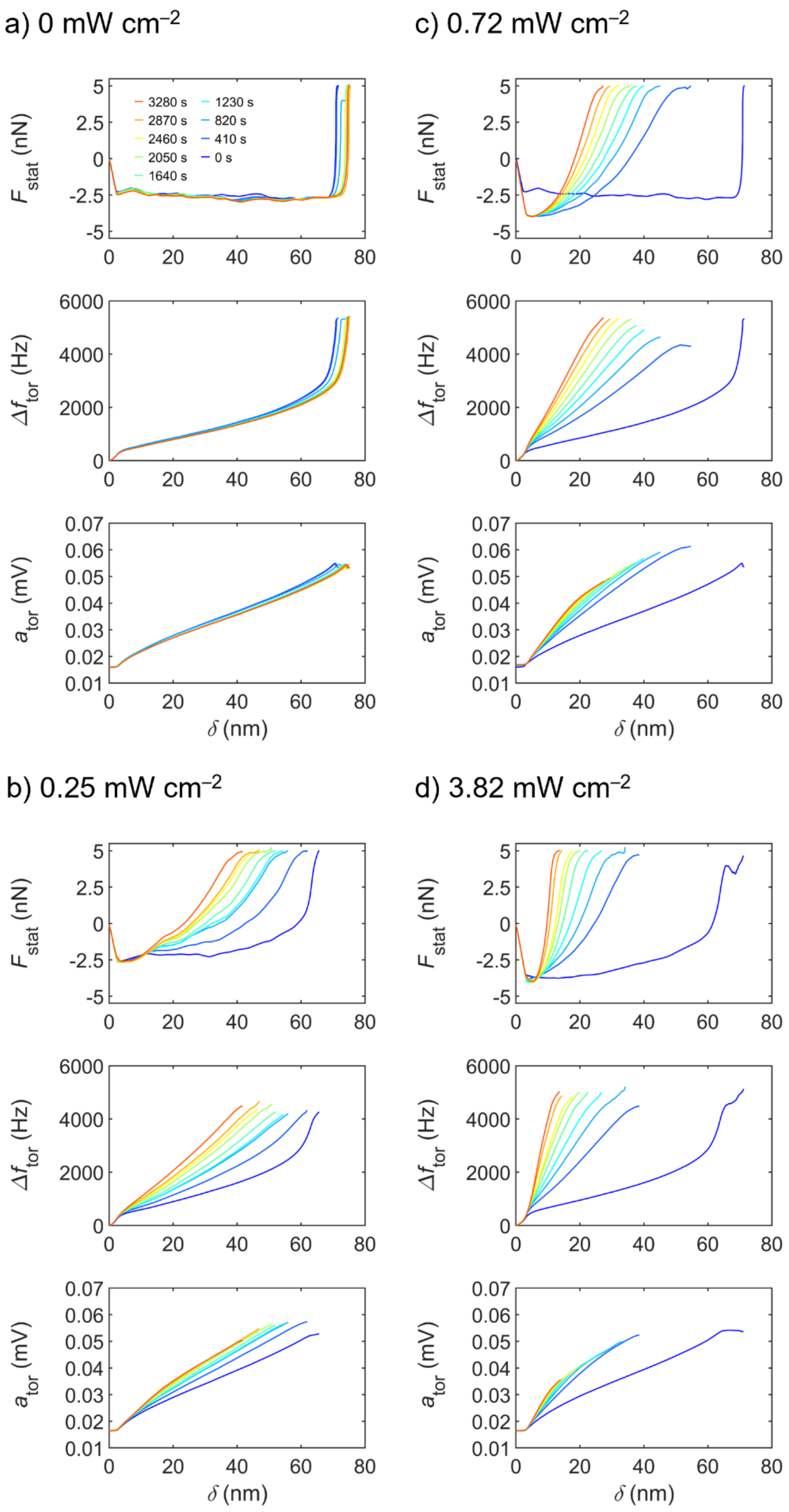

Figure S8: Torsional force spectroscopy observables—vertical static force (F_{stat}), torsional frequency shift (Δf_{tor}), and excitation amplitude (a_{tor})—as functions of indentation depth (δ) and

UV illumination time at varying intensities: a) 0 mW cm^{-2}, b) 0.25 mW cm^{-2}, c) 0.72 mW cm^{-2}, and d) 3.82 mW cm^{-2}. Each curve represents the average of 1024 measurement points from a single force map acquired over an area of 5 × 5 μm^2 at the defined UV illumination time (number of independent samples: $n = 1$). The legend in a) also applies to b)–d). The force maps were recorded using the in situ torsional force spectroscopy configuration with a vertical tip velocity of 2.5 μm s^{-1}, a torsional oscillation amplitude of 0.75 nm, and a set point force of 5 nN.

To further investigate the stiffening behavior of the UV-illuminated PB films, the torsional force spectroscopy observables shown in Figure S8 were converted into mechanical quantities using Equations (1)–(7) from the main text. The resulting curves, plotted as functions of indentation depth, are presented in **Figure S9**. The trends observed for the torsional force spectroscopy observables are reflected in the corresponding mechanical quantities. Specifically, without UV illumination, the four mechanical quantities remain similar (see Figure S9a). Upon UV exposure (see Figure S9b–d), clear differences emerge: with increasing UV illumination time, the torsional tip–sample force, in-plane shear stress, dissipated energy, and storage shear modulus all increase. Notably, the torsional tip–sample force and dissipated energy exhibit a steeper rise at higher UV intensities, indicating accelerated reaction rates of the UV-induced cross-linking process. At the highest UV intensity investigated (3.82 mW cm^{-2}), a distinct peak appears in both the in-plane shear stress and storage shear modulus at an indentation depth of approximately 15 nm as illumination time increases (see Figure S9d).

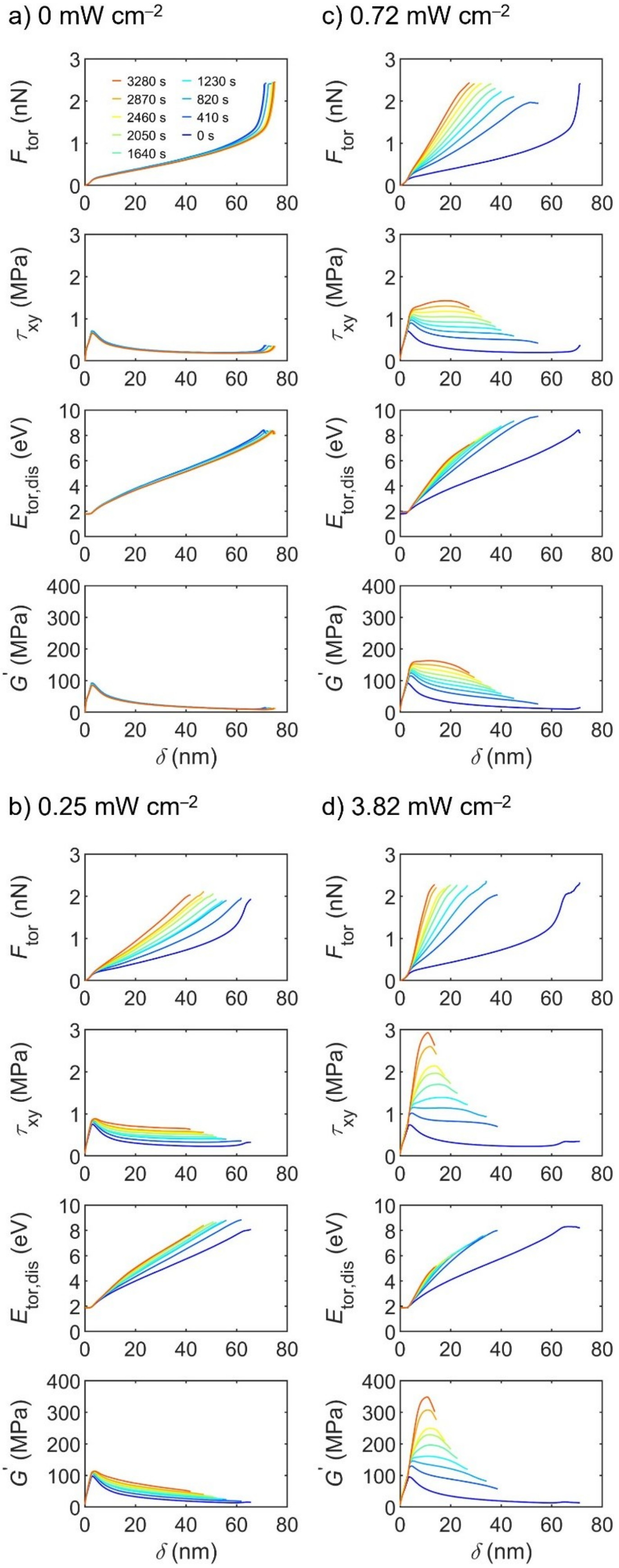

Figure S9: Torsional force spectroscopy mechanical quantities—torsional tip–sample force (F_{tor}), in-plane shear stress (τ_{xy}), torsional dissipated energy ($E_{tor,dis}$), and storage shear modulus

(G')—as functions of indentation depth (δ) and UV illumination time at varying intensities: a) 0 mW cm^{-2}, b) 0.25 mW cm^{-2}, c) 0.72 mW cm^{-2}, and d) 3.82 mW cm^{-2}. Each curve represents the average of 1024 measurement points from a single force map acquired over an area of 5 × 5 μm^2 at the defined UV illumination time (n = 1). The legend in a) also applies to b)–d). The force maps were recorded using the in situ torsional force spectroscopy configuration with a vertical tip velocity of 2.5 μm s^{-1}, a torsional oscillation amplitude of 0.75 nm, and a set point force of 5 nN.

S8. Patterning of PB Film with Aluminum Foil for Ex situ Torsional Force Spectroscopy

To assess the accuracy of the in situ torsional force spectroscopy measurements, complementary ex situ torsional force spectroscopy was performed. Unlike the in situ approach, where measurements are taken during UV illumination, the ex situ method involves performing torsional force spectroscopy after UV exposure of the PB film. The detailed procedure is illustrated in **Figure S10**.

A sharp boundary between UV-illuminated and non-illuminated regions was essential because the illuminated spots were not visible in the DriveAFM camera during in situ experiments. For this purpose, aluminum foil was tightly stretched across the PB film surface and fixed to the sample stage with adhesive tape (see Figure S10a). The UV illumination spot was then defined along the foil edge, indicated by the white dashed rectangle in Figure S10a.

Before UV exposure, the cantilever holder (without cantilever) was mounted on the atomic force microscope, and its approach to the PB film surface was verified at the same height as when the cantilever would be in contact with the film (see Figure S10b). The light beam in

Figure S10b marks the UV illumination spot. Figure S10c shows the camera image of the aluminum foil covering a part of the PB film, with a defect in the lower left corner serving as a reference point for locating the UV-illuminated area after the foil removal. The red dashed square denotes the $100 \times 100\ \mu m^2$ scan frame, which serves as both a scale bar and measurement area.

After positioning, the DriveAFM camera was removed, the UV light source was mounted according to Figure S2 and the illumination was carried out. Following exposure, the UV source was removed, the camera was reinstalled, and the aluminum foil was carefully peeled off to minimize any displacement of the sample, ensuring accurate relocation of the illuminated area.

Finally, the cantilever was mounted, and the camera image in Figure S10d shows the distinct transition between the foil-covered and UV exposed regions (blue brackets). The defect visible in the lower center matches the one in Figure S10c, confirming that the illuminated spot could be easily relocated due to the careful foil removal. The cantilever was positioned at this boundary, and torsional force spectroscopy was performed over a $90 \times 90\ \mu m^2$ area, including both UV exposed and covered regions. The resulting data are presented in **Figure 3** of the main text.

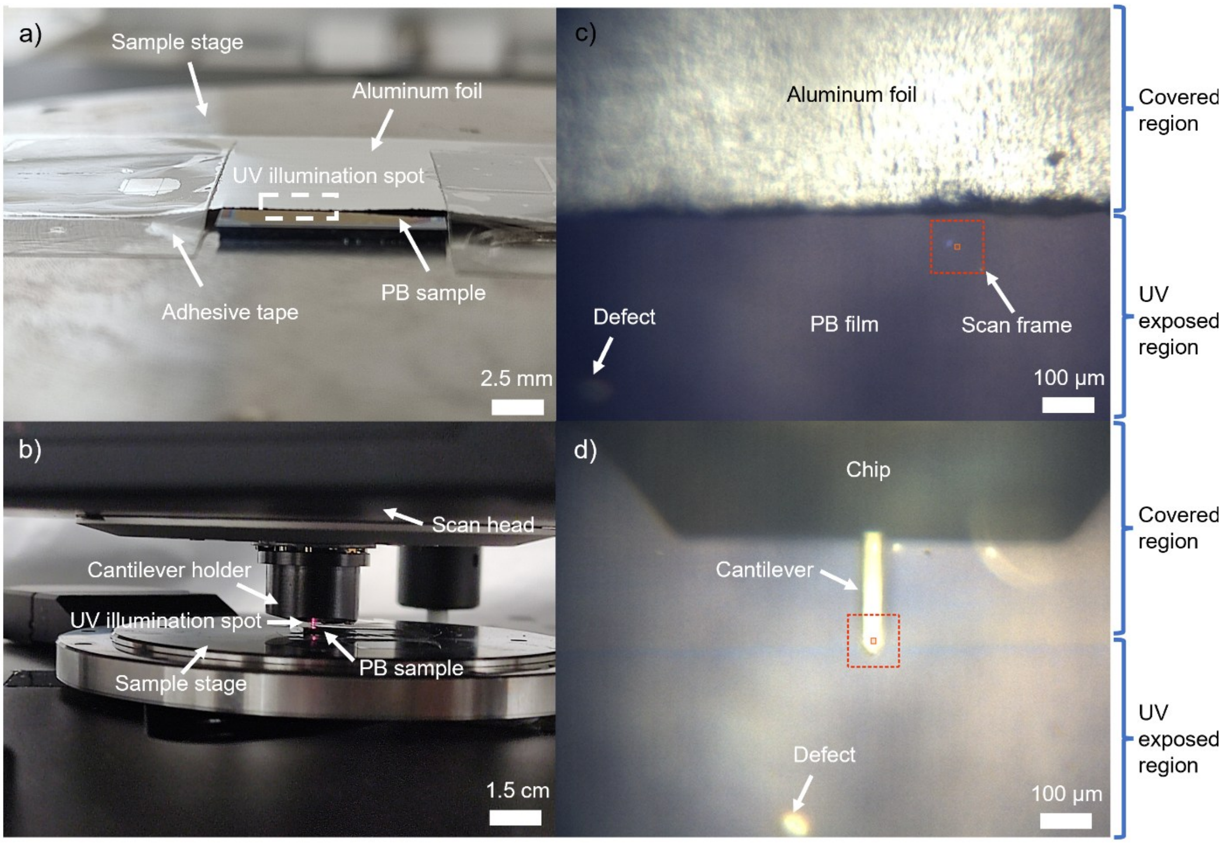

Figure S10: Patterning procedure of PB film for ex situ torsional force spectroscopy: a) PB sample partially covered with aluminum foil, fixed to the sample stage using adhesive tape; b) approach of the AFM scan head with the cantilever holder to the PB film surface (cantilever not yet mounted); c) AFM camera image of the partially covered PB film prior to UV illumination; and d) image after UV illumination, with the aluminum foil removed and the cantilever mounted.

S9. Torsional Force Spectroscopy Observables as Functions of Indentation Depth for In situ and Ex situ measurements

To evaluate the accuracy of the in situ UV torsional force spectroscopy measurements, the torsional force spectroscopy observables obtained under both in situ and ex situ configurations are plotted as functions of indentation depth in **Figure S11**. For the ex situ measurements, the

PB sample was partially covered with aluminum foil during UV illumination (see Figure S10). After illumination, the foil was removed, and torsional force spectroscopy was performed at the transition between foil-covered and UV exposed regions. The corresponding mechanical quantities are presented in Figure 3 of the main text.

The in situ vertical static force curve (blue) in Figure S11a remains in the adhesive regime shorter than the ex situ curve (red), up to an indentation depth of 15 nm. This initial region is likely dominated by liquid meniscus formation upon the AFM tip's first contact with the PB film, probably explaining the adhesive regime.

The transition from the attractive to the repulsive regime occurs at a higher indentation depth for the ex situ curve (≈40 nm) than the in situ curve (≈25 nm). This suggests that the UV-induced cross-linking reactions (see Figure S1) and the associated stiffening may have been less efficient during the ex situ process, possibly due to UV scattering at the aluminum edges or shielding effects of the PB film surface, which could limit UV penetration into the film interior. After this transition, the slopes of the in situ and ex situ curves appear comparable, but the maximum indentation depth is lower for the in situ measurements at the same set point force (5 nN), indicating more efficient stiffening under in situ conditions.

The torsional frequency shift curves in Figure S11b match well up to 15 nm of indentation depth, but deviations increase at greater depths, again suggesting differences in cross-linking efficiency. For the torsional excitation amplitude (see Figure S11c), different starting values are observed, likely due to variations in temperature, relative humidity, and alignment of the detection and CleanDrive lasers on different measurement days. Between 3 and 20 nm the slope of the in situ and ex situ torsional excitation amplitude curves are similar; beyond 20 nm, the in situ curve (blue) exhibits a lower slope than the ex situ curve (red), consistent with more

efficient cross-linking in the in situ configuration, also reflected in the vertical static force and torsional frequency shift trends (see blue curves in Figure S11a and b).

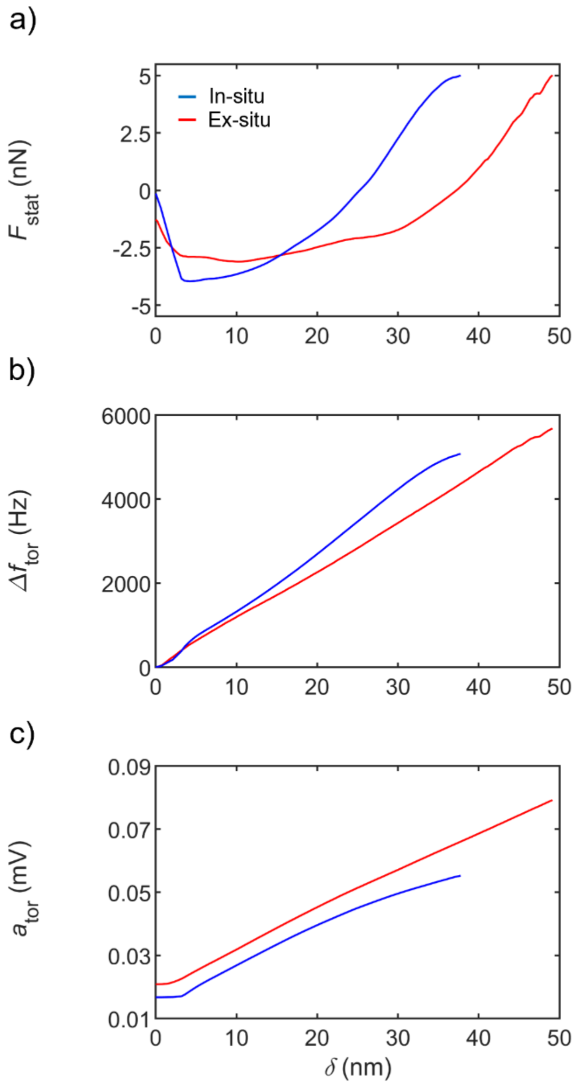

Figure S11: Comparison of torsional force spectroscopy observables obtained from in situ (blue curves) and ex situ (red curves) UV torsional force spectroscopy as functions of indentation depth: a) vertical static force, b) torsional frequency shift, and c) torsional excitation amplitude. The blue curves represent averages of 1024 measurement points acquired over a 5 × 5 μm^2 area (n = 1), whereas the red curves correspond to averages of 100 measurement points collected over a 14 × 14 μm^2 area (n = 1). The respective locations are marked by black dashed frames in the depth profile images in Figure 3e) (in-plane shear stress) and 3f) (torsional dissipated

energy) at an indentation depth of 20 nm. Both in situ and ex situ experiments were performed under otherwise identical conditions, UV illumination for 1640 s at an intensity of 0.72 mW cm^{-2}, a vertical tip velocity of 2.5 μm s^{-1}, a set point force of 5 nN, and a torsional oscillation amplitude of 0.75 nm.

S10. Comparison of Torsional Force Spectroscopy between Polystyrene (PS) Domains within PB Films and Pure PS Film

To identify the origin of the white spots in the depth profile images in **Figure 4**a–c of the main text, we compared torsional force spectroscopy observables and derived mechanical quantities of PB films containing PS with those of a pure PS film as functions of indentation depth **(see Figure S12)**. The pure PS film was prepared using identical spin coating parameters and included an underlying PAM layer. For consistency, PS with the same number-averaged molecular weight (33 kg/mol) and polydispersity index (1.07) dissolved in toluene was used.

The green dashed rectangles in the depth profile images in Figure S12a and b indicate the regions from which the torsional force spectroscopy observables and mechanical quantities were averaged. The resulting curves as functions of indentation depth are presented in Figure S12c–h.

The vertical static force curve of the pure PS film (red curve) shows a short attractive regime up to ≈4 nm indentation depth, slightly shorter than for PB films containing 1 and 2 mg PS (≈5 and 6 nm). The maximum indentation depths (≈4 nm for pure PS and ≈6 nm for PB with PS) are similar at the same set point force of 5 nN. In contrast, the UV-illuminated PB matrix in

Figure S13a exhibits a much higher maximum indentation depth of up to 90 nm for the same set point force, confirming its significantly lower stiffness compared to the white spots.
The torsional frequency shift curves in Figure S12d further support this observation: all three curves exhibit a steep increase up to ≈5 nm and reach a frequency shift of 5000 Hz at roughly this depth, whereas the UV-illuminated PB matrix requires 50 and 90 nm indentation to reach the same value (see Figure S13b).

The torsional excitation amplitude curves in Figure S12e start from different values, likely due to day-to-day variations in temperature, relative humidity, and laser alignment. However, the slopes consistently show a faster increase for the pure PS film than for the PB films with PS, suggesting that UV illumination of the PB films (1640 s) reduced the torsional excitation amplitude response.

The mechanical quantities derived from these observables are shown in Figure S12f–h. The torsional tip–sample force curves (see Figure S12f) rise steeply up to ≈5 nm. The in-plane shear stress curves (see Figure S12g) reach values of up to ≈10 MPa, roughly an order of magnitude higher than those of the UV-illuminated PB matrix (see Figure 4 of the main text). While the curves are similar until ≈3 nm indentation depth, the pure PS film shows about twice the shear stress of the PB films with 1 mg and 2 mg PS at 5 nm, likely reflecting the embedding of PS in the softer PB matrix. A comparable trend appears for the torsional dissipated energy curves (see Figure S12h), which start from different offsets but show a steeper slope for the pure PS film.

Taken together, the strong agreement between the torsional force spectroscopy signatures of the white spots in PB films closely matches those of the pure PS film which confirms that these white spots correspond to PS domains within the PB matrix.

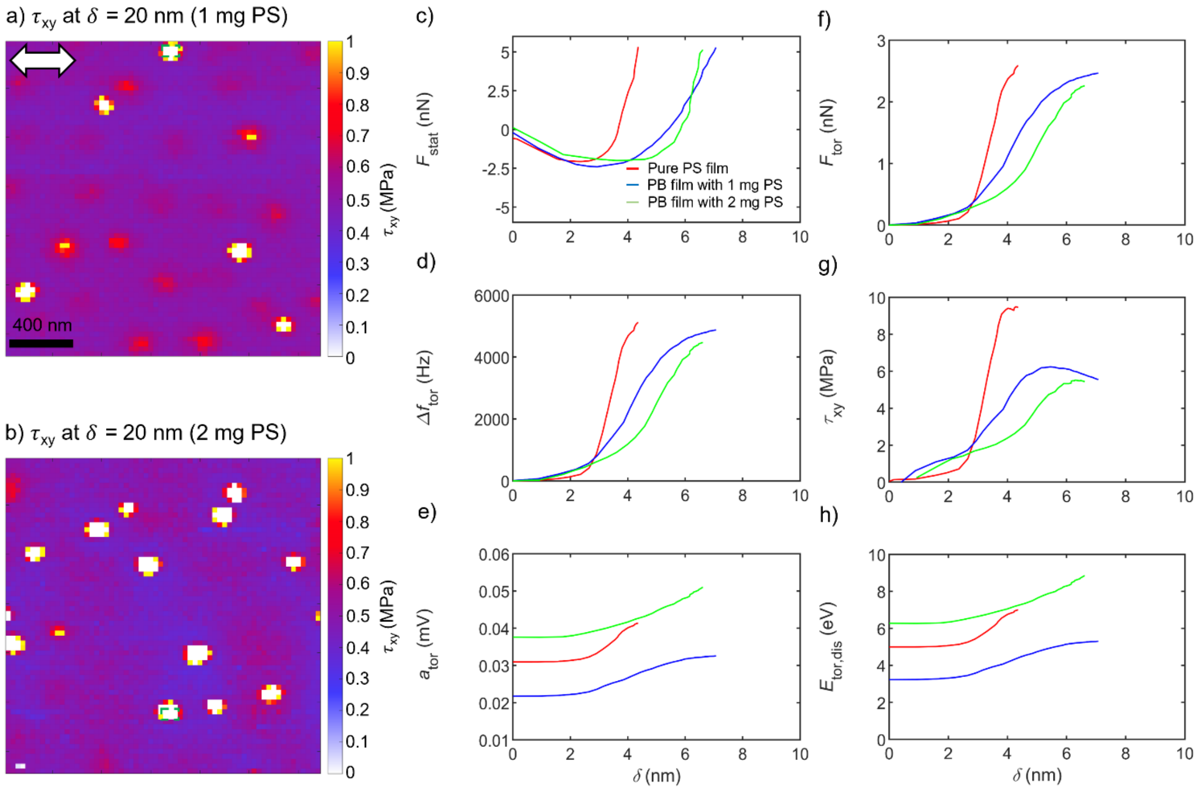

Figure S12: Comparison of torsional force spectroscopy observables and mechanical quantities for a pure PS film and PB films containing constant amounts of DEABP and TRIS with varying PS contents. Depth profile images of in-plane shear stress at an indentation depth of 20 nm for PB films with a) 1 mg PS and b) 2 mg PS. The white double arrow in a) indicates the AFM tip shearing direction; the same scale bar and arrow apply to b). White spots in a) and b) mark endpoints of the depth profile curves at the defined set point force of 5 nN, corresponding to high stiffness of PS domains. Averaged torsional force spectroscopy observables from the regions marked by green dashed rectangles (six points over 93 × 62 nm^2 for PB films (n = 1); 16 points over 5 × 5 μm^2 for the pure PS film (n = 1)): c) vertical static force, d) torsional frequency shift, and e) torsional excitation amplitude. Derived mechanical quantities: f) torsional tip–sample force, g) in-plane shear stress, and h) torsional dissipated energy. The PS film was pristine and non-UV-illuminated; PB films with 1 mg and 2 mg PS were UV-illuminated for 1640 s at an intensity of 0.72 mW cm^{-2}. All measurements were taken with a

vertical tip velocity of 2.5 μm s^{-1}, set point force of 5 nN, and torsional oscillation amplitude of 0.75 nm.

S11. Influence of PS on the Torsional Force Spectroscopy Observables of UV-illuminated PB Films as Functions of Indentation Depth

To investigate the effect of PS on the cross-linking efficiency of the UV-illuminated PB films, torsional force spectroscopy observables were analyzed for PB films containing constant amounts of DEABP and TRIS with varying PS contents, as shown in Figure S13. The corresponding mechanical quantities and depth profile images are presented in Figure 4 of the main text.

The red vertical static force curve in Figure S13a for the PB film without PS remains in the attractive regime up to an indentation depth of approximately 25 nm. Within this range, it exhibits the most negative vertical static force, indicating the strongest attractive interactions due to the absence of rigid PS domains. The transition from the attractive to the repulsive regime for this curve occurs at roughly 40 nm of indentation depth. As the PS content in the PB film increases, this transition shifts to larger indentation depths, and the maximum indentation depth at the same set point force of 5 nN also increases (see blue and green curves in Figure S13a). This behavior indicates decreased stiffening of the PB matrix with increasing PS content.

A similar trend can also be observed for the torsional frequency shift in Figure S13b: higher PS contents result in lower torsional frequency shifts at the same indentation depth, except within the first 5 nm, where capillary forces from the liquid meniscus dominate tip–sample interactions.

The torsional excitation amplitude curves in Figure S13c exhibit different initial values, likely due to variations in temperature, relative humidity, and alignment of the detection and CleanDrive lasers between measurement days, as discussed previously. Overall, the slope of the red curve (PB film without PS) is higher than that of the blue and green curves (PB films with PS), suggesting enhanced irreversible arrangements of the cross-linked PB chains induced under lateral tip motion in the absence of PS.

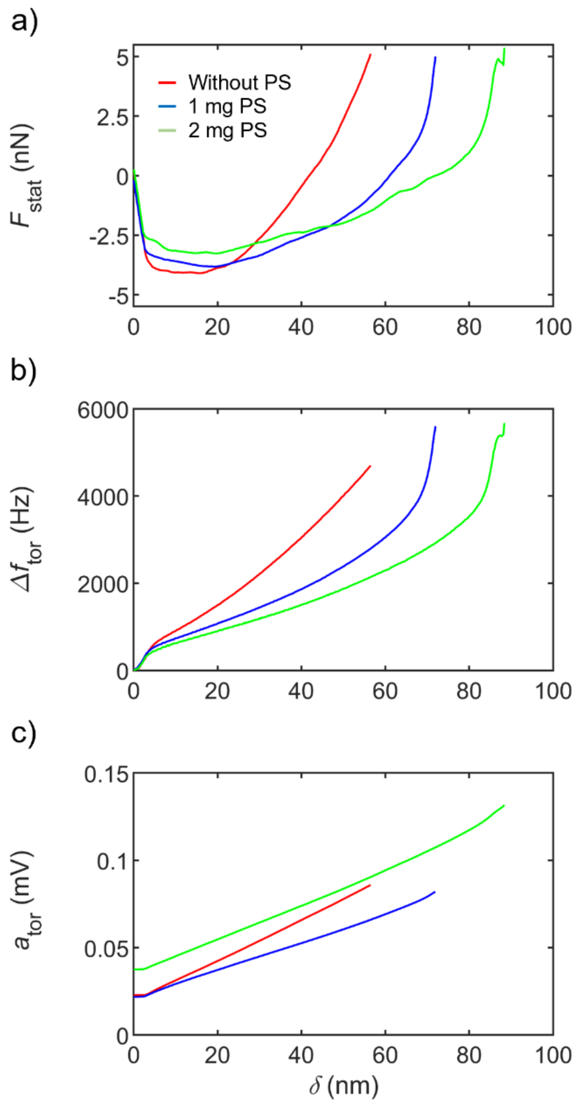

Figure S13: In situ UV torsional force spectroscopy observables as functions of indentation depth for PB films containing constant amounts of DEABP and TRIS but varying PS content: a) vertical static force, b) torsional frequency shift, and c) torsional excitation amplitude. Each

curve represents an average of 25 measurement points acquired over a 156 × 156 nm^2 area (n = 1), with the corresponding locations indicated by black dashed frames in the depth profile images of in-plane shear stress in Figure 4a)–c) at an indentation depth of 20 nm. UV illumination was performed for 1640 s at an intensity of 0.72 mW cm^{-2}. Torsional force spectroscopy mapping was conducted with a vertical tip velocity of 2.5 μm s^{-1}, a set point force of 5 nN, and a torsional oscillation amplitude of 0.75 nm.

S12. X-ray Photoelectron Spectroscopy (XPS) of Pure PB Films and PB Films with DEABP, TRIS, and Varying PS Contents

To investigate the spatial distribution of the cross-linking agent TRIS with addition of PS, XPS spectra were recorded for pristine (non-UV-illuminated) pure PB films as well as PB films containing constant amounts of DEABP and TRIS with varying PS contents **(see Figure S14)**.

The C1s spectra in Figure S14a exhibit a distinct peak at approximately 285 eV. However, this peak cannot be unambiguously assigned to the individual polymers because the C1s binding energy reported for PB (284.74 eV) and PS (284.76 eV) is nearly identical due to their structures containing carbon–carbon double bonds.[8] Consequently, the peaks of PB and PS cannot be distinguished from one another.

Instead, the analysis focuses on signals originating from the photoinitiator (DEABP) and the cross-linking agent (TRIS). Figure S14b provides a magnified view of the C1s spectra on a logarithmic intensity scale for improved peak visualization. A peak at 289.4 eV becomes apparent, which can be attributed to the ester group (O-C=O) of TRIS, since none of the other film components contains this functional group.[9] This peak is clearly visible for the PB film

without PS (green curve), but appears significantly reduced in intensity for the PB film with PS (blue curve), and is absent in the pure PB film lacking both DEABP and TRIS. These findings suggest a redistribution of the cross-linking agent TRIS upon addition of PS, such as vertical phase separation or surface-sensitive segregation effects.

To further verify this interpretation, the S2p spectra were analyzed (see Figure S14c). Distinct doublets, due to spin-orbit splitting of the 2p-orbital, at approximately BE (S $2p_{3/2}$) = 163.5 eV and BE (S $2p_{1/2}$) 164.7 eV are observed for PB films containing DEABP and TRIS (blue and green curves) and can be assigned to the thiol groups (-SH) of TRIS, as no other film component contains sulfur atoms.[9] These peaks are absent in the pure PB film without DEABP and TRIS (see red curve in Figure S14c). Importantly, the intensity of the S2p doublets decreases significantly upon addition of PS (see blue curve in Figure S14c), consistent with the C1s findings (see Figure S14a and b).

Finally, the O1s spectra in Figure S14d reveal distinct double peaks at approximately 532.8 eV and 534.3 eV for the PB film without PS, which correspond to the ester functionalities of TRIS.[10]

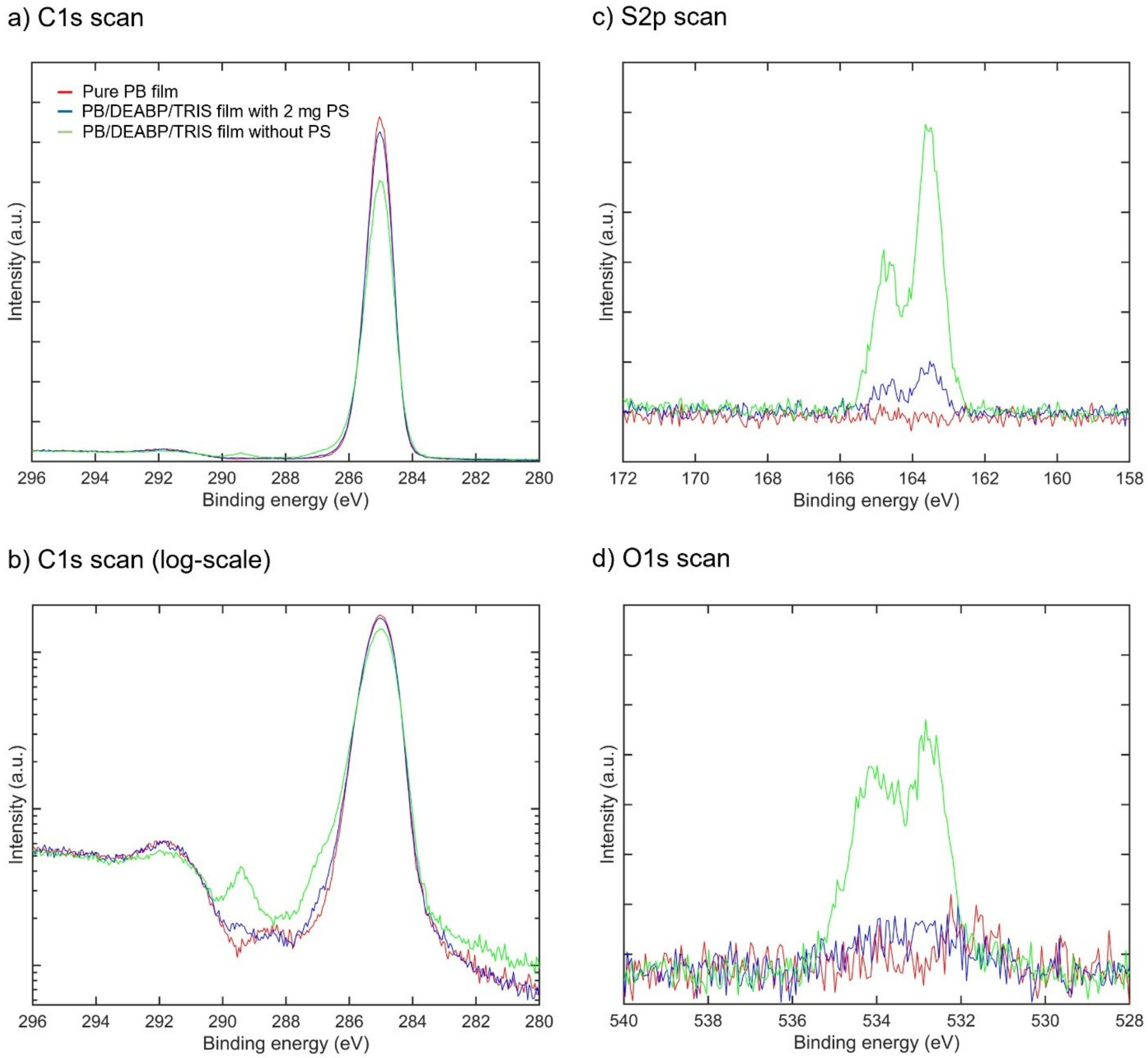

Figure S14: XPS spectra of pristine (non-UV-illuminated) pure PB and PB films containing constant amounts of DEABP and TRIS with varying PS contents: a) C1s scan, b) C1s scan (logarithmic intensity scale), c) S2p scan, and d) O1s scan.

S13. Fourier Transform Infrared Reflection Absorption Spectroscopy (FT-IRRAS) on Pure PB Films and PB Films with DEABP, TRIS, and with/without PS

Since XPS probes only the top ≈10 nm of thin films and did not reveal distinct PS signals, FT-IRRAS measurements were performed to clarify whether the decreases in the thiol and ester signals observed by XPS are restricted to the top-surface region or occur throughout the entire film thickness.

Figure S15 shows FT-IRRAS spectra of a pure PB film (red) and PB films containing constant amounts of DEABP and TRIS without (green) and with 2 mg PS (blue). Two absorption bands exhibit pronounced differences among the samples. The first band at 701 cm^{-1} (see orange rectangle) corresponds to the out-of-plane bending vibration of the aromatic rings and is characteristic for PS.[11] It is clearly visible for the PB film containing 2 mg PS (blue) but absent in the pure PB film (red) and in the PB film without PS (green), confirming the presence of PS domains within the PB film. The second band at 1743 cm^{-1} (see yellow rectangle) is assigned to the ester C=O vibration of TRIS.[12] This band is prominent in the PB film without PS (green) but becomes markedly reduced in the PB film with PS (blue), while it does not appear in the pure PB film (red).

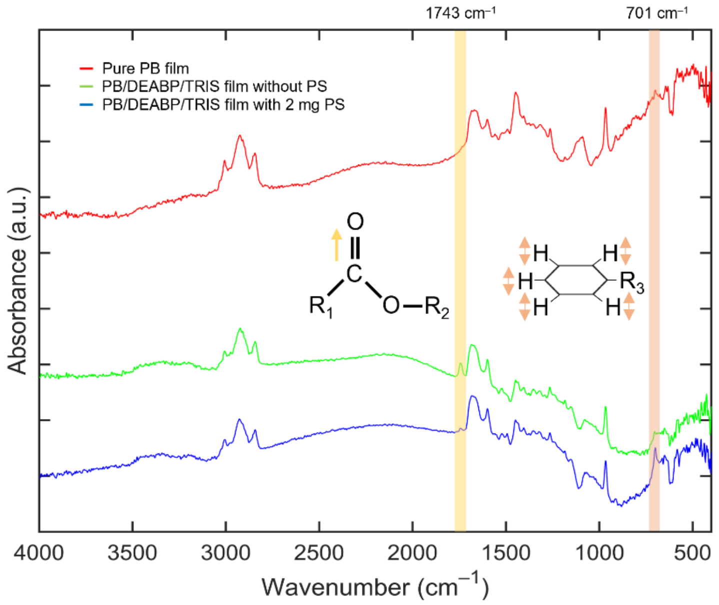

Figure S15: FT-IRRAS spectra of pristine (non-UV-illuminated) pure PB and PB films containing constant amounts of DEABP and TRIS with and without PS. Characteristic absorption bands at 701 cm^{-1} (PS) and 1743 cm^{-1} (TRIS ester group) are highlighted by semi-transparent rectangles at their respective wavenumber positions. R_1, R_2, and R_3 denote generic organic substituents and are used here to simplify the representation of the relevant functional groups.

S14. Discussion of the Spatial Distribution of TRIS within PB Films upon Addition of PS

Based on the XPS and FT-IRRAS results, several scenarios for the spatial distribution of the TRIS cross-linking agent within the PB film upon addition of PS can be considered and are discussed below.

In the reference case without PS (see **Figure S16**a), TRIS is assumed to be homogenously distributed across the film thickness. Therefore, the O1s signal in XPS as well as the absorption

band at 1743 cm^{-1}, representing the ester group of TRIS, appear without any distinct attenuation.

In the first scenario of the PS-containing PB films (see Figure S16b), vertical phase separation can occur due to addition of PS. This may lead to a decrease of the O1s signal in XPS compared to the reference case without PS due to a depletion of the TRIS molecules within the volume of the near-surface and the limited probing depth of XPS ($\leq$ 10 nm). In contrast, FT-IRRAS probes throughout the entire film thickness. Therefore, the signal intensity of the ester absorption band should not considerably change with respect to the reference case because the overall concentration of TRIS along the film thickness should remain constant.

In the second scenario of the PS-containing PB films (see Figure S16c), the addition of PS may induce a surface aggregation of TRIS, which can be explained by differences in molecular size and polarity between TRIS, PB, and PS. Low molecular weight molecules, such as TRIS, tend to segregate toward interfaces to minimize the free surface energy and maximize the configurational entropy, as shown in other polymer systems.[13] Furthermore, PS and PB are rather hydrophobic polymers with low polarity in contrast to TRIS, which exhibits ester and thiol groups, resulting in a high polarity and interfacial activity.

Within the ultrathin layer of TRIS at the surface of the PB film, the TRIS molecules may arrange in a preferred orientation, with the thiol groups aligned to the interface between PB film and air. This can result in an attenuated O1s signal in XPS even in the presence of TRIS. FT-IRRAS, probing the entire PB film thickness, might detect a weak absorption band of the ester group at 1743 cm^{-1} due to its detection limit.

In the third scenario (see Figure S16d), TRIS aggregates at the interface of the PB film and the underlying PAM layer, attributed to the high polarity of its amine (-NH) groups. Consequently, no O1s signal indicating the ester group of TRIS should be detectable in XPS. However, a small peak is detected in our XPS measurements, excluding this scenario.

Comparing the measured XPS and FT-IRRAS spectra with the different scenarios of the PS-containing PB films, the scenario of the surface aggregation of TRIS represents the most probable case, indicating the decreased signals from the ester group arises from spatial redistribution of TRIS rather than from inhibition of cross-linking reactions.

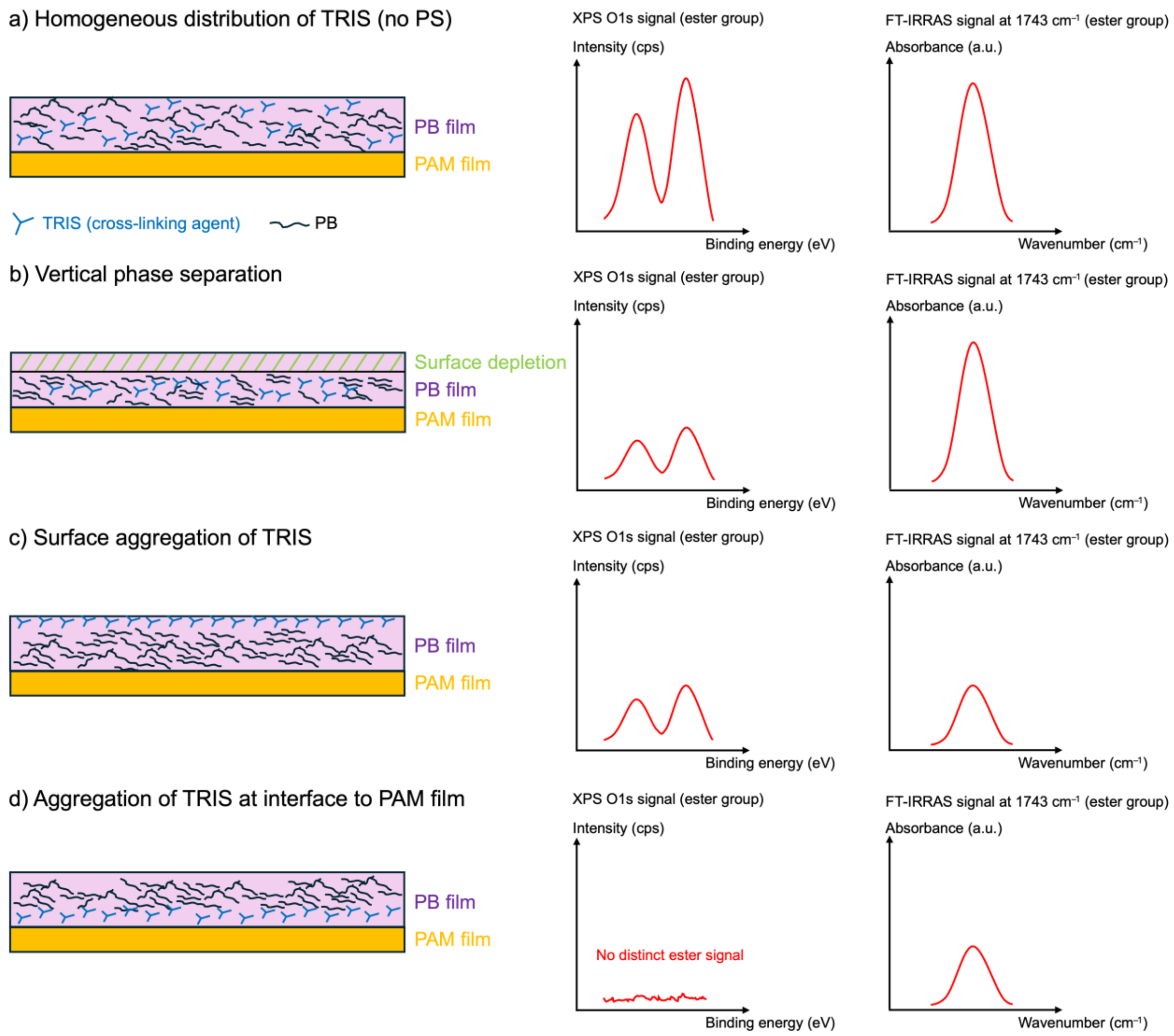

Figure S16: Schematic representation of distribution of cross-linking agent (TRIS) molecules within PB films a) without PS and b)–d) hypothetical distributions of TRIS in PB films containing PS, including b) vertical phase separation, c) surface aggregation of TRIS, and d) aggregation of TRIS at the interface of the PB film and the underlying PAM layer. For each scenario, the expected qualitative XPS and FT-IRRAS signal intensities are depicted.

References

(1) Decker, C.; Nguyen Thi Viet, T. High-Speed Photocrosslinking of Thermoplastic Styrene–Butadiene Elastomers. *J. Appl. Polym. Sci.* **2000**, *77*, 1902–1912. DOI: 10.1002/1097-4628(20000829)77:9<1902::AID-APP6>3.0.CO;2-6.

(2) Green, C. P.; Lioe, H.; Cleveland, J. P.; Proksch, R.; Mulvaney, P.; Sader, J. E. Normal and Torsional Spring Constants of Atomic Force Microscope Cantilevers. *Rev. Sci. Instrum.* **2004**, *75*, 1988–1996. DOI: 10.1063/1.1753100.

(3) Hoffer, M.; Petersein, F.; Dehnert, M.; Lintner, T. A.; Dietz, C. Anisotropic Subsurface Inter- and Intramolecular Properties of Heterogeneous Polymers Revealed by Torsional Force Spectroscopy. *Langmuir* **2025**, *41*, 14360–14372. DOI: 10.1021/ACS.LANGMUIR.5C01423.

(4) Datta, S.; Stocek, R.; Naskar, K. Influence of Ultraviolet Radiation on Mechanical Properties of a Photoinitiator Compounded High Vinyl Styrene–Butadiene–Styrene Block Copolymer. *Polymers* **2021***, 13*, 1287. DOI: 10.3390/POLYM13081287.

(5) Rettler, E. F.-J.; Rudolph, T.; Hanisch, A.; Hoeppener, S.; Retsch, M.; Schubert, U. S.; Schacher, F. H. UV-Induced Crosslinking of the Polybutadiene Domains in Lamellar Polystyrene-Block-Polybutadiene Block Copolymer Films – An In-Depth Study. *Polymer* **2012**, *53*, 5641–5648. DOI: 10.1016/J.POLYMER.2012.09.054.

(6) Okamura, H.; Yamagaki, M.; Nakata, K. Analysis of Network Structures in Thiol-Ene UV Curing System Using Reworkable Resins. *Polymers* **2019**, *11*. DOI: 10.3390/POLYM11010005.

(7) Dehnert, M.; Magerle, R. 3D Depth Profiling of the Interaction between an AFM Tip and Fluid Polymer Solutions. *Nanoscale* **2018**, *10*, 5695–5707. DOI: 10.1039/C8NR00299A.

(8) Beamson, G.; Briggs, D. *High Resolution XPS of Organic Polymers: The Scienta ESCA300 Database*; John Wiley & Sons Ltd, 1992; pp 65–72.

(9) Duan, Q.; Wang, Y.; Chen, S.; Miao, M.; Chen, S.; Zhang, D. Functionalized Carbon Nanotube Films by Thiol-Ene Click Reaction. *Appl. Surf. Sci.* **2019**, *486*, 144–152. DOI: 10.1016/J.APSUSC.2019.05.011.

(10) López, G. P.; Castner, D. G.; Ratner, B. D. XPS O 1s Binding Energies for Polymers Containing Hydroxyl, Ether, Ketone and Ester Groups. *Surf. Interface Anal.* **1991**, *17*, 267–272. DOI: 10.1002/SIA.740170508.

(11) Hanulikova, B.; Capkova, T.; Antos, J.; Urbanek, M.; Urbanek, P.; Sevcik, J.; Kuritka, I. Temperature Dependence of Vibrational Motions of Thin Polystyrene Films by Infrared Reflection-Absorption Spectroscopy: A Single Measurement Tool for Monitoring of Glass Transition and Temperature History. *Polym. Test.* **2021**, *101*, 107305. DOI: 10.1016/J.POLYMERTESTING.2021.107305.

(12) Necolau, M. I.; Biru, E. I.; Olaret, E.; Iovu, H. Multi-Functional Hybrid Terpolymer Thermosets Based on Thiols Bio-Based Epoxy and Benzoxazine Monomers. *Polymers* **2025**, *17*, 2389. DOI: 10.3390/POLYM17172389.

(13) Yurtsever, A.; Gigler, A. M.; Dietz, C.; Stark, R. W. Frequency Modulated Torsional Resonance Mode Atomic Force Microscopy on Polymers. *Appl. Phys. Lett.* **2008**, *92*, 143103. DOI: 10.1063/1.2907498/166312.
